# Metabolic profiling of *Fargesia yunnanensis* shoots across developmental stages and responses to preharvest jasmonic acid treatment

**DOI:** 10.1016/j.fochx.2026.104669

**Published:** 2026-10-24

**Authors:** Jiaxin Liu, Yingdan Yan, Wanli Shi, Fangwei Zhu, Bo Zheng, Lingfeng Li, Ye Chen, Jiaxin Ran, Lixia Yu, Huijin Fan, Jialong Pei, Shuguang Wang

**Affiliations:** a Biological Research and Utilization Innovation Team in Bamboo Resources of Yunnan Province, Southwest Forestry University, Kunming 650224, China; b Science and Technology Innovation Team of National Forestry and Grassland Administration, Southwest Forestry University, Kunming 650224, China; c Faculty of Bamboo and Rattan, Southwest Forestry University, Kunming 650031, China

**Keywords:** *Fargesia yunnanensis*, Bamboo shoots, Preharvest treatment, Jasmonic acid, Nutrients

## Abstract

Bamboo shoot quality is influenced by developmental stage and phytohormones, but stage-dependent metabolic changes and the effects of preharvest hormone treatment remain unclear. Here, untargeted and targeted metabolomics were used to profile *Fargesia yunnanensis* shoots across growth stages and under jasmonic acid (JA) treatment. The most pronounced metabolic changes occurred during early shoot development. Shoot buds accumulated bitter amino acids and phenolics. In contrast, edible-stage shoots showed high moisture content and relatively low levels of cellulose and lignin. Aged shoots had higher levels of cell wall components and altered phenolic acid profiles. Responses differed between the two tested JA concentrations. Low JA reduced soluble sugars, amino acids, and phenolics, whereas high JA increased most soluble sugars, amino acids, rutin, and major phenolic acids, but decreased most flavonoids and increased lignin. Overall, shoot quality was closely associated with developmental stage, and preharvest JA application requires comprehensive evaluation.

## Highlights

• Metabolic differences were greatest between shoot buds and edible-stage shoots.
• Edible-stage shoots had higher moisture and lower lignin contents.
• Low JA promoted elongation and total soluble sugars but reduced protein content.
• High JA increased selected sugars, amino acids, and rutin contents.
• High JA also increased lignin contents and reduced many other flavonoids content.

## 1 Introduction

Bamboo shoots are the tender buds of bamboo, and are regarded as an ideal nutritional resource because they are rich in high-quality protein, carbohydrates, minerals, and vitamins, while being low in fat (Wang, Chen, et al., 2020). Their dietary fiber promotes digestion and weight management, and bioactive components such as polysaccharides and alkaloids have potential therapeutic value. In addition, the abundant phenolic compounds in bamboo shoots also exhibit favorable biological activities, including antioxidant, anti-inflammatory, and blood glucose-regulation effects (Wang, Chen, et al., 2020; Ma et al., 2024). Differences in nutrient content and secondary metabolite synthesis (such as lignin, flavonoids, and tannins) at different stages of bamboo shoots not only affect their flavor and nutritional value but also determine the optimal harvest time (Huang et al., 2025). Bamboo shoots are rapidly growing harvestable organs that initiate elongation immediately after emergence and undergo active sugar metabolism, cell wall remodeling, and progressive lignification. Previous studies have reported rapid internodal elongation in *Fargesia yunnanensis* shoots and substantial growth in *Phyllostachys edulis* within 24 h during the rapid growth phase, accompanied by secondary cell wall deposition and lignification (Wang, Pei, et al., 2020; Chen et al., 2022). However, excessive lignification alters cell wall composition and nutrient- and flavor-related components, potentially affecting the texture and edible quality of bamboo shoots. Therefore, understanding changes in nutrients and metabolites across developmental stages is essential for elucidating quality formation in bamboo shoots. Nevertheless, integrated analyses of nutritional components, cell wall composition, and metabolite dynamics during the development of *F. yunnanensis* shoots remain limited.

Preharvest pretreatment refers to deliberate intervention in the growth, development, and physiological metabolism of fruits or vegetables through nutrient management, environmental regulation, or the application of exogenous active substances before harvest to improve quality at harvest and subsequently influence postharvest storability and shelf life (Zhao et al., 2024). In recent years, many studies have demonstrated that preharvest hormone treatment not only regulates crop yield and appearance quality, but also significantly affects the accumulation of nutrients and functional components in fruits and vegetables, including vitamins, amino acids, carotenoids, flavonoids, and phenolic compounds (Gong et al., 2022). Jasmonates (JAs) are endogenous plant signaling molecules that regulate diverse physiological processes, including defense responses, growth inhibition, and secondary metabolite biosynthesis (Ghorbel et al., 2021). In preharvest regulation of fruits and vegetables, exogenous JAs have been widely used to improve quality, with methyl jasmonate (MeJA) being the most commonly applied form. Previous studies have established that foliar MeJA application increased soluble sugar and relative moisture contents in *Brassica napus*, with implications for sensory quality (Ahmadi et al., 2018). Moreover, exogenous MeJA promoted phenolic accumulation in *Lactuca sativa* (Almeida et al., 2025), increased sugars and flavonoids contents and antioxidant capacity in *Rubus idaeus* (Wang & Zheng, 2005), and enhanced yield and phenolic-linked antioxidant activity in *Cynara cardunculus* (Martínez-Esplá et al., 2017). Although recent studies have mainly focused on MeJA, exogenous jasmonic acid (JA) has also been used directly as a preharvest treatment in some studies. Preharvest JA application improved sugar-acid balance, anthocyanin accumulation, and harvest quality in *Punica granatum* (Hussein et al., 2023), reduced preharvest fruit drop in seedless grape (*Vitis vinifera*) more effectively than MeJA (Fidelibus et al., 2022), and influenced sugar accumulation and postharvest shelf life in *Carica papaya* (Devarakonda et al., 2020). These studies indicate that JA and MeJA can affect growth and quality-related traits in various fruits and vegetables, but their effects may vary depending on plant species, treatment substance, and treatment conditions.

JA and MeJA can interconvert in plants. JA is converted to MeJA by jasmonic acid carboxyl methyltransferase (JMT), whereas MeJA is hydrolyzed to JA by MeJA esterase (MJE) (Seo et al., 2001). JA can be further converted to JA-Ile, which participates in COI1-JAZ-mediated jasmonate signaling (Wang et al., 2021). Although preharvest JA treatment has been investigated in fruits and vegetables, its application and physiological effects in bamboo shoot production remain poorly understood. In particular, the effects of preharvest JA treatment on shoot growth, cell wall components, and metabolite accumulation remain unclear, as do the concentration-dependent responses of these traits. Therefore, this study investigated the effects of different JA concentrations on the growth, cell wall components, and metabolic composition of *F. yunnanensis* shoots. The findings may help elucidate the potential role of JA in shoot growth and quality formation and inform the development of preharvest treatments for bamboo shoots.

*F. yunnanensis*, an alpine bamboo species mainly distributed in Sichuan and Yunnan provinces of China, is valued locally for its tender shoots (Yu et al., 2021). This study investigated *F. yunnanensis* shoots by combining physiological and biochemical analyses with untargeted and targeted metabolomics to compare their nutritional components and metabolic profiles across developmental stages and assess physiological and metabolic changes following 24 h of preharvest JA treatment. The study aims to provide evidence to inform further evaluation of preharvest JA treatment and bamboo shoot composition.

## 2 Materials and methods

### 2.1 Materials

The shoot buds of *F. yunnanensis* develop on underground rhizomes near the parent bamboo culms, beginning to form between April and June before emerging and elongating. This study compared metabolic differences across three developmental stages, with samples collected when the shoots met predefined morphological criteria. Based on growth position, shoot height, degree of lignification, and internode elongation status, the samples were classified into three natural developmental stages: bamboo shoot buds (Sb), referring to intact underground buds attached to rhizomes before emergence, edible-stage bamboo shoots (ES, 30–50 cm in height), and aged bamboo shoots (AS, 100 cm in height), whose first internode had completed elongation (Fig. 1A). In this study, underground shoot buds that had not yet emerged and the first three aboveground internodes of emerged shoots were selected as experimental materials. All samples were collected from the bamboo garden at Southwest Forestry University between June and September 2023 under clear, rain-free conditions. Nine bamboo clumps were selected, and samples representing all three developmental stages were collected from these clumps. Samples from every three clumps were pooled to form one biological replicate, yielding three biological replicates per developmental stage. Each biological replicate was measured in technical replicate, and the technical measurements were averaged before statistical analysis. After collection, the samples were divided into two portions. One portion was used immediately for moisture content determination, and the other was stored in an ultra-low-temperature freezer for subsequent physiological, biochemical, untargeted metabolomic, and targeted metabolomic analyses.

**Fig. 1:**
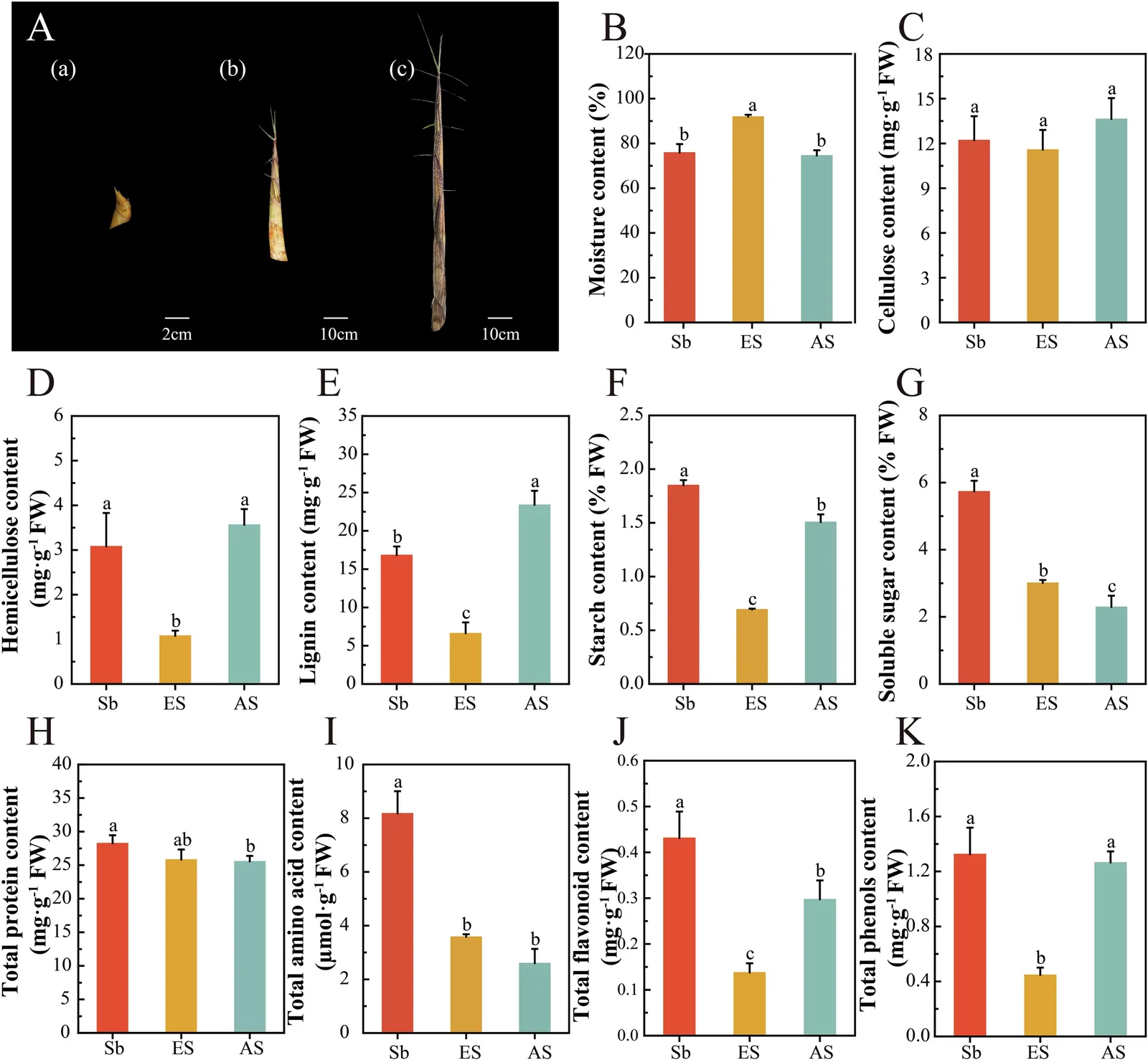
**Changes in nutrient composition of *F. yunnanensis* shoots at different growth stages.** (A) Growth of *F. yunnanensis* shoots at different growth stages. (a) Shoot buds, Bar = 2 cm. (b) Bamboo shoots of 30–50 cm in height, Bar = 10 cm. (c) Aged bamboo shoots whose first internode has completed elongation growth, Bar =10 cm. (B—K) Changes in nutrient composition at different stages. (B) Moisture content. (C) Cellulose content. (D) Hemicellulose content. (E) Lignin content. (F) Starch content. (G) Soluble sugar content. (H) Protein content. (I) Amino acid content. (J) Flavonoid content. (K) Total phenol content. Data are presented as mean ± SD from three biological replicates (*n* = 3). Statistical significance was assessed using one-way ANOVA followed by Duncan's multiple comparison test. Different lowercase letters indicated significant differences (*p* < 0.05) among growth stages. Sb, shoot buds; ES, normal edible bamboo shoots (30–50 cm in height); AS, aged bamboo shoots, whose first internode had completed elongation growth.

### 2.2 Methods

#### 2.2.1 Determination of moisture content

The shoot samples were heated at 105 °C for 15 min and then oven-dried at 60 °C to constant weight. The moisture content was calculated as follows: Moisture content (%) = [(FW-DW)/FW] × 100, where FW and DW represent fresh weight and dry weight, respectively.

#### 2.2.2 Determination of cell wall component content

Cellulose, hemicellulose, and lignin contents were determined using BC4280, BC4440, and BC4200 assay kits (Beijing Solarbio Science & Technology Co., Ltd., Beijing, China), respectively. The shoot samples were used to determine cellulose content, whereas samples dried to constant weight were used to determine hemicellulose and lignin content. Sample preparation and assays were performed according to the corresponding kit instructions. Cellulose and hemicellulose contents were determined spectrophotometrically at 620 and 540 nm, respectively. Lignin content was determined using the acetyl bromide method. After sequential extraction and centrifugation, the insoluble residue was retained for the acetyl bromide reaction, and absorbance was measured at 280 nm.

#### 2.2.3 Determination of soluble sugar and starch content

Soluble sugar and starch contents were determined according to Okahisa et al. (2006). 0.5 g samples were ground in liquid nitrogen, extracted with deionized water, and centrifuged. The supernatant was used for soluble sugar determination, while the precipitate was re-extracted with deionized water and 9.2 mol/L perchloric acid for starch determination. Both carbohydrates were quantified colorimetrically using the phenol‑sulfuric acid method.

#### 2.2.4 Determination of total protein and total amino acid content

The total protein content was determined using the assay kit (CW0014S, Jiangsu Cowin Biotech Co., Ltd., Jiangsu, China). The samples were homogenized in 1 × PBS solution. 100 μL of the sample was added to a test tube, and the working solution was added according to the manufacturer's instructions. The mixture was incubated in a 37 °C water bath for 30 min, then cooled to room temperature, and the absorbance was measured at 560 nm using a spectrophotometer (UV-8000, MWTASH, Shanghai, China) within 3–5 min. The total protein concentration was calculated.

The total amino acid content was determined using the BC1570 assay kit (Beijing Solarbio Science & Technology Co., Ltd., Beijing, China). Fresh sample (0.1 g) was weighed, and the required working solutions were added in sequence according to the instructions. The absorbance at 570 nm was measured, and the total amino acid content was calculated.

#### 2.2.5 Determination of total flavonoid and total phenolic content

The contents of total flavonoid and total phenols were detected using BC1335 and BC1340 assay kits (Beijing Solarbio Science & Technology Co., Ltd., Beijing, China), respectively. For the determination of these two substances, samples (0.1 g) were weighed in EP tubes, and then the extracts in the respective kits were added for ultrasonic extraction. After that, reagent addition and reaction were carried out according to the instructions. The contents of total flavonoid and total phenols were determined by visible spectrophotometry at 470 nm and 760 nm, respectively.

#### 2.2.6 Untargeted metabolomic analysis

Untargeted metabolomic analysis of bamboo shoot samples was performed using liquid chromatography-tandem mass spectrometry (LC-MS/MS). Fresh samples (0.02 g) were placed in 2 mL EP tubes, and 600 μL of pre-cooled methanol (containing 4 μg/mL 2-chloro-*L*-phenylalanine) was added. After brief vortexing, glass beads were added, and the mixture was ground at 60 Hz for 90 s using a bead beater, followed by sonication at room temperature for 15 min. The samples were centrifuged at 12,000 rpm (4 °C) for 10 min, and the supernatant was filtered through a 0.22 μm PTFE filter before LC-MS analysis. A pooled quality control (QC) sample was prepared by mixing equal volumes of extracts from all biological samples to monitor LC–MS analytical stability. QC samples were processed and analyzed using the same procedures as the biological samples.

The chromatographic conditions were set according to Zelena et al. (2009), and the mass spectrometric conditions according to Want et al. (2013). Raw data were converted to mzXML format using ProteoWizard MSConvert. Peak detection, filtering, retention time correction, and alignment were performed using the XCMS package in R. Peak area data were then normalized for subsequent statistical analysis, and metabolite annotation was carried out using the HMDB, MassBank, LipidMaps, mzCloud, KEGG, and self-built material library.

#### 2.2.7 Targeted analysis of soluble sugars

Fresh samples (50 mg) were mixed with zirconium beads and 1 mL of aqueous methanol, then subjected to intermittent shaking (50 Hz), vortexing (1 min), and sonication (5 min). After centrifugation at 12,000 rpm for 5 min, 400 μL of the supernatant was vacuum-concentrated to dryness. The residue was reconstituted in 80 μL of 20 mg/mL methoxyamine hydrochloride in pyridine and incubated at 60 °C for 60 min. Subsequently, derivatization was performed with 100 μL of BSTFA-TMCS (99:1) at 70 °C for 90 min. After centrifugation at 12,000 rpm for 5 min, 100 μL of the supernatant was collected for analysis. Sample separation was performed using an Agilent 7890 gas chromatograph (Agilent Technologies, Santa Clara, CA, USA) coupled with a Pegasus BT time-of-flight mass spectrometer, equipped with a DB-5MS capillary column (30 m × 0.25 mm × 0.25 μmol·L^−1^; Agilent Technologies, Santa Clara, CA, USA). Helium served as the carrier gas at a flow rate of 3 mL/min. The injection volume was 1 μL. Mass spectra were recorded in the range of *m*/*z* 85–500 at a scan rate of 10 spectra/s. A total of 18 soluble sugars were selected as target analytes for detection and quantification (Table S1). Target compounds were identified based on the retention times and characteristic quantifier ions of authentic standards and quantified using the external standard method. The calibration curves, linear ranges, and limits of quantification (LOQ) are provided in Table S1. Representative GC–MS selected ion monitoring (SIM) chromatograms of the target carbohydrate standards are shown in Fig. S1.

#### 2.2.8 Targeted analysis of free amino acids

A fresh samples (15 mg) were weighed into EP tubes, and steel balls and 1000 μL of pre-cooled extraction solvent (acetonitrile/methanol/water, 2:2:1, *v*/v/v) containing an isotope-labeled internal standard mixture were added. The samples were vortexed for 30 s, ground at 40 Hz for 4 min, and ultrasonicated in an ice-water bath for 5 min. The grinding and ultrasonication were repeated three times. The samples were then kept at −40 °C for 1 h and centrifuged at 12,000 rpm for 15 min at 4 °C. The supernatant was diluted tenfold, vortexed for 30 s, and transferred to injection vials for UHPLC-MS/MS analysis. The amino acid contents were determined using an Agilent 1290 Infinity II UHPLC system (Agilent Technologies, Santa Clara, CA, USA). Separation of the target analytes was performed on a Waters ACQUITY UPLC BEH Amide column (100 × 2.1 mm, 1.7 μmol·L^−1^; Waters, Milford, MA, USA). The mobile phases were 1% formic acid in water (A) and 1% formic acid in acetonitrile (B). The column temperature was maintained at 35 °C, the autosampler temperature at 4 °C, and the injection volume was 1 μL. Mass spectrometric data were acquired in multiple reaction monitoring (MRM) mode, with quantifier and qualifier transitions established for each amino acid. Amino acid contents were calculated using calibration curves with isotope-labeled internal standard correction. The calibration curves were constructed using the peak area ratios of the target amino acids to their corresponding isotope-labeled internal standards as response values, rather than using the peak areas of the target analytes directly for external calibration. A total of 25 amino acids were targeted for detection and quantification. The MRM parameters, retention times of authentic standards, calibration ranges, coefficients of determination (R^2^), limits of detection (LOD), limits of quantification (LOQ), recoveries, and relative standard deviations (RSD) for the 25 amino acids are provided in Table S2. Representative extracted ion chromatograms of selected target amino acids in the standard solution and sample are shown in Fig. S2.

#### 2.2.9 Targeted analysis of phenolic compounds

Fresh samples (100 mg) were weighed into EP tubes, and 500 μL of 80% aqueous methanol containing 0.2% vitamin C was added. The samples were vortexed and ultrasonicated at room temperature for 30 min, then centrifuged at 12,000 rpm for 10 min. The resulting supernatant was transferred to injection vials for LC-MS analysis. Throughout the analytical procedure, the samples were maintained in an autosampler at 4 °C. Data acquisition was performed using a Vanquish UHPLC system coupled with a Q Exactive high-resolution mass spectrometer (Thermo Fisher Scientific, Waltham, MA, USA). Chromatographic separation of the target analytes was achieved on a Waters HSS T3 column (50 × 2.1 mm, 1.8 μmol·L^−1^; Waters, Milford, MA, USA). The mobile phases consisted of ultrapure water containing 0.1% formic acid (A) and acetonitrile containing 0.1% formic acid (B). The column temperature was maintained at 40 °C, the flow rate was 0.3 mL/min, and the injection volume was 2 μL. Quantification of phenolic compounds was performed using the external standard method. A total of 40 phenolic compounds were included in the method for detection and quantification, including 21 flavonoids, 17 phenolic acids, and 2 other phenolic compounds (resveratrol and gossypol) (Table S3), Information on the authentic standards, R^2^, LOD and LOQ for the 40 target phenolic compounds is provided in Table S3. Total ion chromatograms of the standard solution and bamboo shoot samples are shown in Fig. S3.

#### 2.2.10 Effect of exogenous JA on the nutritional composition of bamboo shoots

Lanolin was used as the carrier for JA application. JA was dissolved in absolute ethanol (1 g in 2 mL) and diluted to prepare working solutions. For each formulation, the required aliquot of JA working solution was brought to a total volume of 111.1 μL with absolute ethanol and incorporated into 30 g of lanolin melted in a warm water bath. The mixture was thoroughly mixed and cooled to room temperature before application. Nominal JA concentrations of 10, 50, 100, and 150 μmol·L^−1^ corresponded to 2.22, 11.11, 22.22, and 33.34 μg JA per g of lanolin, respectively. The JA-free vehicle formulation was prepared using the same procedure, with 111.1 μL of absolute ethanol added to 30 g of lanolin without JA. Each bamboo shoot, approximately 50 cm in height, received a single application of 25 g of the corresponding formulation to the sheath surface in its middle-to-upper portion.

For concentration screening, shoots received formulations corresponding to nominal JA concentrations of 0, 10, 50, 100, or 150 μmol·L^−1^, with the 0 μmol·L^−1^ group receiving the JA-free vehicle formulation. Shoot height was measured at 24 h intervals for five consecutive days. Based on the growth responses, 10 and 150 μmol·L^−1^ JA were selected for subsequent experiments, and samples were collected 24 h after a single application. Untreated shoots sampled at 0 h (C0) served as the baseline reference, whereas shoots receiving the JA-free vehicle formulation and sampled after 24 h (C24) served as the time-matched vehicle control. Shoots receiving 10 μmol·L^−1^ JA (L24) or 150 μmol·L^−1^ JA (H24) were also sampled after 24 h. Each group comprised three biological replicates, yielding a total of 12 biological samples for nutritional measurements and untargeted and targeted metabolomic analyses.

#### 2.2.11 Data analysis

All data were statistically analyzed using Microsoft Excel 2019. One-way analysis of variance (ANOVA) followed by multiple comparisons was performed using SPSS Statistics 21.0 (IBM Corp., Armonk, NY, USA), and figures were generated using Origin 2021 software (OriginLab Corporation, Northampton, MA, USA). Untargeted metabolomic data were further analyzed by orthogonal partial least squares-discriminant analysis (OPLS-DA), and differentially accumulated metabolites (DAMs) were identified based on variable importance in projection (VIP) values. Metabolites with VIP > 1, |log_2_FoldChange| > 1, and *p* < 0.05 were considered DAMs. All experiments were conducted with three biological replicates, and data are presented as the mean ± standard deviation (SD). Principal component analysis (PCA), Venn diagrams, volcano plots, and differential metabolite classification plots were generated using the Majorbio Cloud Platform (Mu et al., 2024). KEGG pathway enrichment analysis, DA-score analysis, and correlation analysis were performed using R version 4.4.2 (R Foundation for Statistical Computing, Vienna, Austria).

## 3 Results

### 3.1 Changes in metabolites of bamboo shoots at different growth stages

The nutritional components of *F. yunnanensis* shoots changed significantly across different growth stages. As development progressed, moisture content initially increased and then decreased, and was significantly higher at the edible bamboo shoot (ES) stage than at the shoot bud (Sb) stage and aged bamboo shoot (AS) stage (Fig. 1B). Cellulose, hemicellulose, and lignin contents all decreased initially and then increased, with the lowest contents at the ES stage (Fig. 1C-E).

Carbohydrate contents in *F. yunnanensis* shoots also changed significantly across different growth stages (Fig. 1F-G). Starch and soluble sugar contents were significantly higher at the Sb stage than at the ES stage and AS stage. Across developmental stages, the starch content decreased significantly during bamboo shoot formation but increased significantly at the aged stage, whereas soluble sugar content decreased significantly throughout development.

Similar to the changes in soluble sugar content, both total protein and total amino acid contents exhibited a declining trend during shoot development (Fig. 1H-I). Total protein content was the highest at the Sb stage and lowest at the AS stage, with a significant difference between these two stages. However, no significant difference was observed between the ES stage and the other two stages (Fig. 1H). Total amino acid content was significantly higher at the Sb stage than at both the ES and AS stages, whereas no significant difference was observed between the latter two stages (Fig. 1I).

In addition, total flavonoid and total phenolic contents also showed significant variation during shoot development. Similar to patterns observed for cell wall components and starch, both total flavonoid and total phenolic contents decreased significantly initially and then increased significantly, reaching their lowest levels at the ES stage (Fig. 1J-K). The lower nutrient levels at the ES stage might be related to both nutrient consumption during the rapid growth and the relatively high moisture content at this stage.

### 3.2 Untargeted metabolomic analysis of bamboo shoots at different growth stages

The analytical stability of the untargeted metabolomics data was first evaluated. The total ion chromatograms of the QC samples showed good overall overlap in both positive and negative ion modes, with consistent retention times and signal profiles of the major chromatographic peaks (Fig. S4A,B). Based on QC sample peak areas, 69.7% and 66.2% of the detected features had RSDs below 30% in positive and negative ion modes, respectively (Fig. S4C,D), indicating good analytical stability.

Principal component analysis (PCA) of the detected metabolites showed that PC1 and PC2 explained 48.14% and 21.80% of the total variance, respectively (Fig. 2A). The PCA score plot showed tight clustering of biological replicates and clear separation among developmental stages, showing within-group similarity and between-stage differences in metabolic profiles. In both positive and negative ion modes, the OPLS-DA models for all pairwise comparisons showed R^2^Y values of 0.9995–0.9999 and leave-one-out cross-validation Q^2^ values of 0.9266–0.9718 (Table S4), indicating good model fit and predictive performance (Fig. S5).

Based on metabolite annotation results, a total of 684 differential metabolites were identified (Fig. 2B), of which 375 were shared between Sb vs ES and Sb vs AS comparisons. Sb vs ES showed the most differential metabolites, whereas ES vs AS showed the fewest, indicating that the largest number of differential metabolites occurred in the Sb versus ES comparison. Across the comparisons, the numbers of upregulated and downregulated metabolites ranged from 54 to 306 and from 110 to 250, respectively (Fig. 2C; Tables S5-S7).

**Fig. 2:**
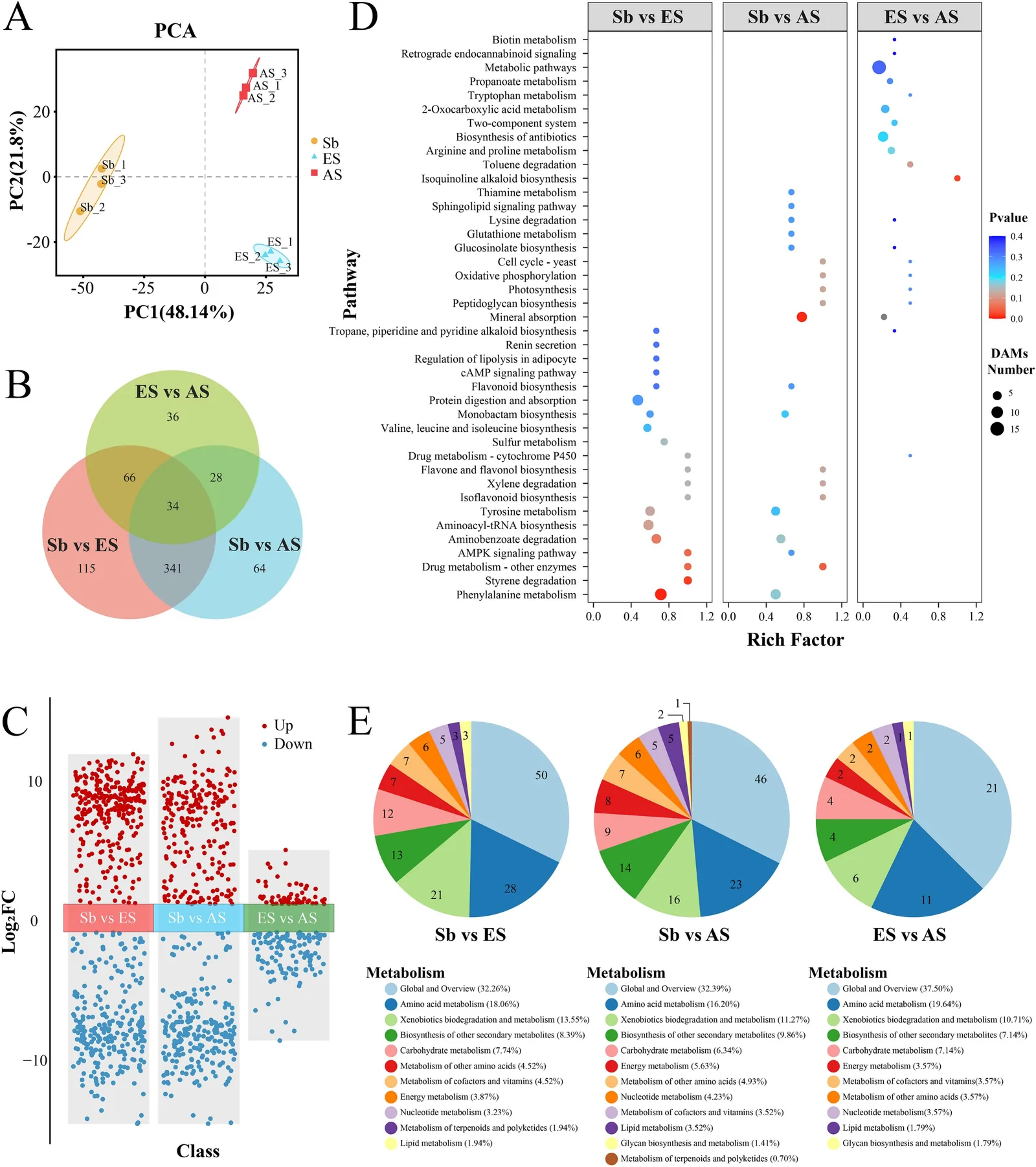
**Untargeted metabolomics analysis of *F. yunnanensis* shoots at different growth stages.** (A) Principal component analysis (PCA) score plot. PC1 and PC2 explained 48.14% and 21.80% of the total variation, respectively, indicating significant metabolic differences among developmental stages. (B) Venn diagram of differential metabolites (DAMs). (C) Statistical analysis of differentially accumulated metabolites. Red dots represented up-regulated metabolites; blue dots represented down-regulated metabolites. (D) KEGG enrichment bubble plot of DAMs across three comparison groups. Bubble size represented the number of metabolites, and color intensity represented the *p*-value. (E) Classification of differential metabolites. Sb, shoot buds; ES, normal edible bamboo shoots (30–50 cm in height); AS, aged bamboo shoots, whose first internode had completed elongation growth. (For interpretation of the references to color in this figure legend, the reader is referred to the web version of this article.)

To further characterize the functions of DAMs, KEGG pathway enrichment analysis was performed for the three comparisons (Fig. 2D). From the Sb stage to the ES stage, DAMs were mainly enriched in amino acid metabolism pathways, including phenylalanine metabolism, tyrosine metabolism, and valine, leucine and isoleucine biosynthesis, as well as in secondary metabolite biosynthesis pathways such as isoflavonoid biosynthesis, flavone and flavonol biosynthesis, and flavonoid biosynthesis. In contrast, from the ES stage to the AS stage, DAMs were mainly enriched in growth-related amino acid metabolism pathways (arginine and proline metabolism, tryptophan metabolism, and lysine degradation) and energy-related pathways (photosynthesis, oxidative phosphorylation, 2-oxocarboxylic acid metabolism, and propanoate metabolism).

In addition, classification of differential metabolites based on the untargeted metabolomic data showed that the differential metabolites in *F. yunnanensis* shoots were mainly assigned to 12 categories (Fig. 2E), including global and overview maps, amino acid metabolism, xenobiotics biodegradation and metabolism, biosynthesis of other secondary metabolites, carbohydrate metabolism, energy metabolism, metabolism of other amino acids, nucleotide metabolism, metabolism of cofactors and vitamins, lipid metabolism, glycan biosynthesis and metabolism, and metabolism of terpenoids and polyketides. Among these categories, metabolites related to amino acid metabolism, secondary metabolite biosynthesis, and carbohydrate metabolism accounted for a relatively high proportion of the total differential metabolites. Overall, the nutrients showing significant changes across different developmental stages were mainly enriched in three major groups, namely soluble sugars, amino acids, and phenolic compounds (flavonoids and phenolic acids). Therefore, targeted metabolomic analysis was performed to further characterize changes in these metabolite classes across the stages of Sb, ES and AS.

### 3.3 Targeted metabolomic analysis of bamboo shoots at different growth stages

#### 3.3.1 Targeted analysis of soluble sugars

Based on the targeted metabolomic analysis, a total of 18 soluble sugars were detected in *F. yunnanensis* shoots at different developmental stages. Among them, raffinose, sucrose, fructose, and glucose were the predominant sugars (Fig. 3A). Raffinose, sucrose, and fructose contents were the highest at the Sb stage, whereas glucose content reached its highest level at the AS stage. In contrast, these sugars were present at relatively low levels at the ES stage (Fig. 3A).

**Fig. 3:**
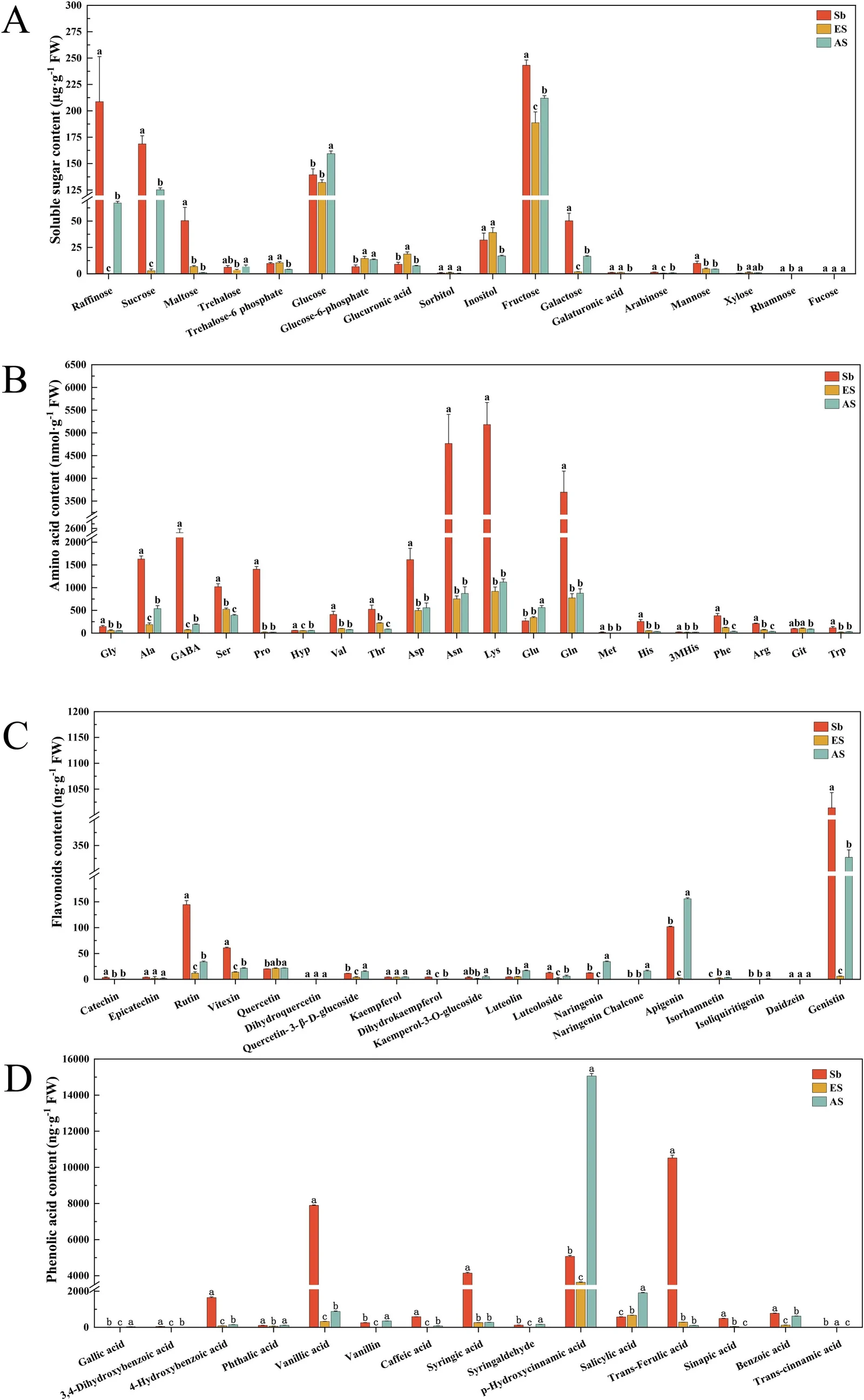
**Changes in nutrient contents of *F. yunnanensis* shoots at different growth stages based on targeted metabolomics analysis.** (A) Soluble sugar content. (B) Amino acid content. (C) Flavonoid content. (D) Phenolic acid content. Data are presented as mean ± SD from three biological replicates (*n* = 3). Statistical significance was assessed using one-way ANOVA followed by Duncan's multiple comparison test. Different lowercase letters indicated significant differences (*p* < 0.05) among growth stages. Sb, shoot buds; ES, normal edible bamboo shoots (30–50 cm in height); AS, aged bamboo shoots, whose first internode had completed elongation growth.

The contents of most soluble sugars were higher in shoot buds than in bamboo shoots and aged bamboo shoots (Fig. S6A). The contents of the majority of soluble sugars, including raffinose, sucrose, trehalose, glucose, fructose, galactose, arabinose, and rhamnose, significantly decreased initially and then partially recovered during bamboo shoot development (Fig. S6B-E, G, L, M, O, R). Only trehalose and glucose contents were more abundant in aged bamboo shoots than in shoot buds. The contents of some other soluble sugars increased initially and then decreased during development, with peak levels at the bamboo shoot stage (Fig. S6A). Among these, levels of glucose-6-phosphate, glucuronic acid, and xylose were significantly higher at the bamboo shoot stage (Fig. S6H, I, Q), whereas trehalose-6-phosphate, sorbitol, inositol, and galacturonic acid levels were slightly higher (Fig. S6F, J, K, N). In contrast, maltose, mannose, and fucose contents gradually decreased during bamboo shoot development, with particularly pronounced decreases in maltose and mannose (Fig. S6D, P, S).

#### 3.3.2 Targeted analysis of free amino acids

Targeted amino acid metabolomics identified 20 amino acids in *F. yunnanensis* shoots at different developmental stages (Fig. 3B), including 6 essential amino acids (Lys, Phe, Thr, Trp, Val, and His). Among these, Asp, Asn, Lys, and Gln were the most abundant (Fig. 3B).

Except for Cit and Glu, the remaining amino acids were more abundant in shoot buds than in bamboo shoots or aged bamboo shoots (Fig. S7A). As shoots aged, the contents of Gly, Ser, Pro, Val, Thr, His, Phe, and Arg decreased, particularly Ser, Thr, Phe, and Arg, which declined significantly (Fig. S7B, E, F, H, I, P, R, S). Differently, the contents of Ala, GABA, Hyp, Asp, Asn, Lys, Gln, 3MHis, and Trp decreased initially and then increased during development, with Ala, Hyp, and GABA showing the most pronounced fluctuations (Fig. S7C, D, G, J-L, N, Q, U). Additionally, the content of Glu increased progressively during bamboo shoot development, reaching significantly higher levels in aged shoots than in shoot buds or bamboo shoots (Fig. S7M), while Cit content showed the highest values in bamboo shoots (Fig. S7T).

#### 3.3.3 Targeted analysis of phenolic compounds

A total of 19 flavonoids were detected in *F. yunnanensis* shoots at different developmental stages (Fig. 3C), among which genistin, apigenin, rutin, vitexin, and quercetin were the main components. A few flavonoids were most abundant in shoot buds, including catechin, epicatechin, rutin, vitexin, dihydrokaempferol, luteoloside, and genistin (Fig. S8A). Among these, epicatechin decreased gradually during shoot development, whereas the other six compounds generally decreased initially and then increased, with rutin, vitexin, dihydrokaempferol, luteoloside, and genistin showing the most pronounced fluctuations (Fig. S8B-E, J, L, T). Most flavonoids were more abundant in aged bamboo shoots (Fig. S8A). Specifically, the contents of quercetin and isorhamnetin increased continuously during aging (Fig. S8F, Q), whereas the contents of quercetin-3-O-glucoside, kaempferol, kaempferol-3-O-glucoside, luteolin, naringenin, apigenin, and isoliquiritigenin decreased initially and then increased during shoot aging (Fig. S8H, I, L-N, P, R).

A total of 15 phenolic acids were identified in *F. yunnanensis* shoot buds and bamboo shoots (Fig. 3D), with p-hydroxycinnamic acid, vanillic acid, trans-ferulic acid, and syringic acid identified as the main components. The contents of 3,4-dihydroxybenzoic acid, 4-hydroxybenzoic acid, vanillic acid, caffeic acid, syringic acid, trans-ferulic acid, sinapic acid, and benzoic acid were significantly higher in shoot buds than in bamboo shoots or aged bamboo shoots (Fig. S9C, D, F, H, I, M-O). Among these, 3,4-dihydroxybenzoic acid, 4-hydroxybenzoic acid, vanillic acid, syringic acid, caffeic acid, and benzoic acid decreased initially from the Sb stage to the ES stage and then increased during the AS stage, whereas trans-ferulic acid and sinapic acid decreased gradually, with sinapic acid exhibiting the most pronounced decline (Fig. S9M, N). In contrast, trans-cinnamic acid content was highest in bamboo shoots, followed by shoot buds, and lowest in aged bamboo shoots (Fig. S9P). In addition, except for salicylic acid, which increased constantly and significantly during bamboo shoot development, the other phenolic compounds, including gallic acid, phthalic acid, vanillin, syringaldehyde, and p-hydroxycinnamic acid, decreased significantly from the Sb stage to the ES stage and then increased during the AS stage (Fig. S9B, E, G, J-L).

Additionally, targeted analysis detected gossypol and resveratrol among the developmental-stage samples (Fig. S10). Gossypol content increased significantly during bamboo shoot aging (Fig. S10A), whereas resveratrol was detected only in aged bamboo shoots (Fig. S10B).

### 3.4 Effects of preharvest JA treatment on growth and metabolite accumulation of *F. yunnanensis* shoots

#### 3.4.1 Effects of preharvest JA treatment on bamboo shoot height

To explore the effect of preharvest JA treatment at different concentrations on the growth of *F. yunnanensis* shoots, we recorded shoot height changes over 5 consecutive days (Fig. 4A and S11). Different concentrations of exogenous JA had significantly different effects on *F. yunnanensis* shoot growth. Compared with the control, 10 μmol·L^−1^ JA significantly promoted shoot height growth, but this effect gradually weakened as the concentration increased, with significant growth inhibition at 150 μmol·L^−1^. Therefore, 10 μmol·L^−1^ and 150 μmol·L^−1^ were selected as low and high concentrations for preharvest JA treatment to evaluate their effects on postharvest metabolite accumulation in *F. yunnanensis* shoots.

#### 3.4.2 Effects of preharvest JA treatment on metabolites in *F. yunnanensis* shoots

JA trestment significantly affected the moisture content of *F. yunnanensis* shoots (Fig. 4B). After 24 h, low JA concentration significantly reduced moisture content compared with both untreated shoots (0 h) and lanolin vehicle control at 24 h. In contrast, the shoots treated with high-concentration JA treatment showed higher moisture contents than those in untreated shoots (0 h) and shoots treated with low JA concentration, but significantly lower moisture content than the lanolin vehicle control at 24 h.

**Fig. 4:**
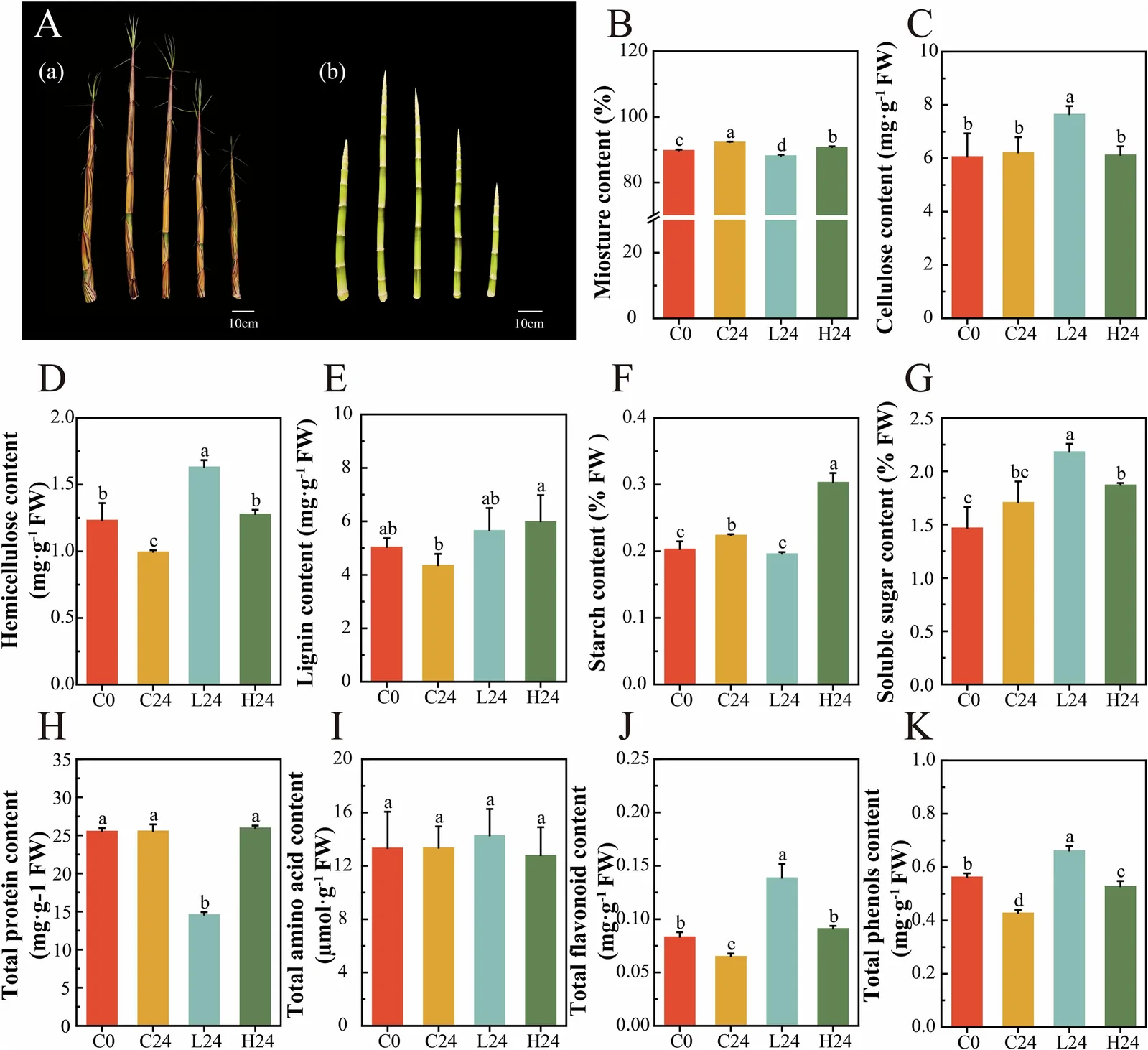
**Growth and changes in nutrient composition of *F. yunnanensis* shoots after application of different JA concentrations.** (A) Growth of *F. yunnanensis* shoots after 5 days of treatment with different JA concentrations. (a) Before shoot sheath removal; (b) After shoot sheath removal. From left to right: 0, 10, 50, 100, and 150 μmol·L^−1^ JA treatment. (B—K) Nutrient composition of *F. yunnanensis* shoots after treatment with different JA concentrations. (B) Moisture content. (C) Cellulose content. (D) Hemicellulose content. (E) Lignin content. (F) Starch content. (G) Soluble sugar content. (H) Protein content. (I) Amino acid content. (J) Flavonoid content. (K) Total phenol content. Data are presented as mean ± SD from three biological replicates (*n* = 3). Statistical significance was assessed using one-way ANOVA followed by Duncan's multiple comparison test. Different lowercase letters indicated significant differences (*p* < 0.05) among treatments. C0, untreated bamboo shoots (0 h); C24, bamboo shoots treated with lanolin (vehicle control) for 24 h; L24, bamboo shoots treated with low concentration of JA (10 μmol·L^−1^) for 24 h; H24, bamboo shoots treated with high concentration of JA (150 μmol·L^−1^) for 24 h.

Unlike moisture content, the low concentration of JA significantly increased cellulose and hemicellulose contents but only slightly increased lignin content in *F. yunnanensis* shoots compared with the control (24 h) (Fig. 4C-E). In contrast, the high concentration of JA slightly reduced cellulose content, while increased lignin content.

Exogenous JA also significantly affected carbohydrate accumulation in *F. yunnanensis* shoots. The low concentration of JA slightly reduced starch content but significantly increased soluble sugar content, whereas high JA concentration significantly enhanced the accumulation of both in bamboo shoots (Fig. 4F-G).

Relative to the changes in cell wall components and non-structural carbohydrate contents, the preharvest JA treatment exerted only minor effects on total protein and total amino acid contents in *F. yunnanensis* shoots (Fig. 4H-I), except that low JA concentration significantly reduced total protein content, while high JA concentration had no significant effect on protein or amino acid levels.

Flavonoid and total phenol assays showed that both flavonoid and total phenol contents decreased significantly during *F. yunnanensis* shoot growth (Fig. 4J-K). Compared with the control (24 h), preharvest JA treatment for 24 h significantly increased both flavonoid and total phenol contents. Notably, the decrease in moisture content under low JA concentration might also have contributed to the higher metabolites levels.

#### 3.4.3 Untargeted metabolomic analysis of *F. yunnanensis* shoots after preharvest JA treatment

The overlaid total ion chromatograms and peak-area RSD distributions of QC samples in positive and negative ion modes for the exogenous JA treatment experiment are shown in Fig. S12. Metabolic features with QC RSDs below 30% accounted for 76.2% and 81.7% of the detected features in positive and negative ion modes, respectively (Fig. S12C,D). PCA based on the combined data matrix from both ion modes showed that biological replicates within the same group tended to cluster together, whereas samples from different treatment groups were separated, indicating differences in their overall metabolic profiles (Fig. 5A). The OPLS-DA model fitting statistics and permutation test results for each pairwise comparison are presented in Table S8 and Fig. S13, respectively.

Based on the screening criteria, 242 differential metabolites mapped to KEGG pathways were identified (Fig. 5B). Compared with the control (normally grown shoots at 24 h), low JA concentration treatment yielded 123 differential metabolites, including 58 up-regulated and 65 down-regulated. High JA concentration treatment exhibited the highest number of differential metabolites (167), with 41 up-regulated and 126 down-regulated. Compared with the low JA concentration treatment, the high JA concentration treatment resulted in 29 up-regulated and 100 down-regulated metabolites (Fig. 5C; Tables S9–S11).

**Fig. 5:**
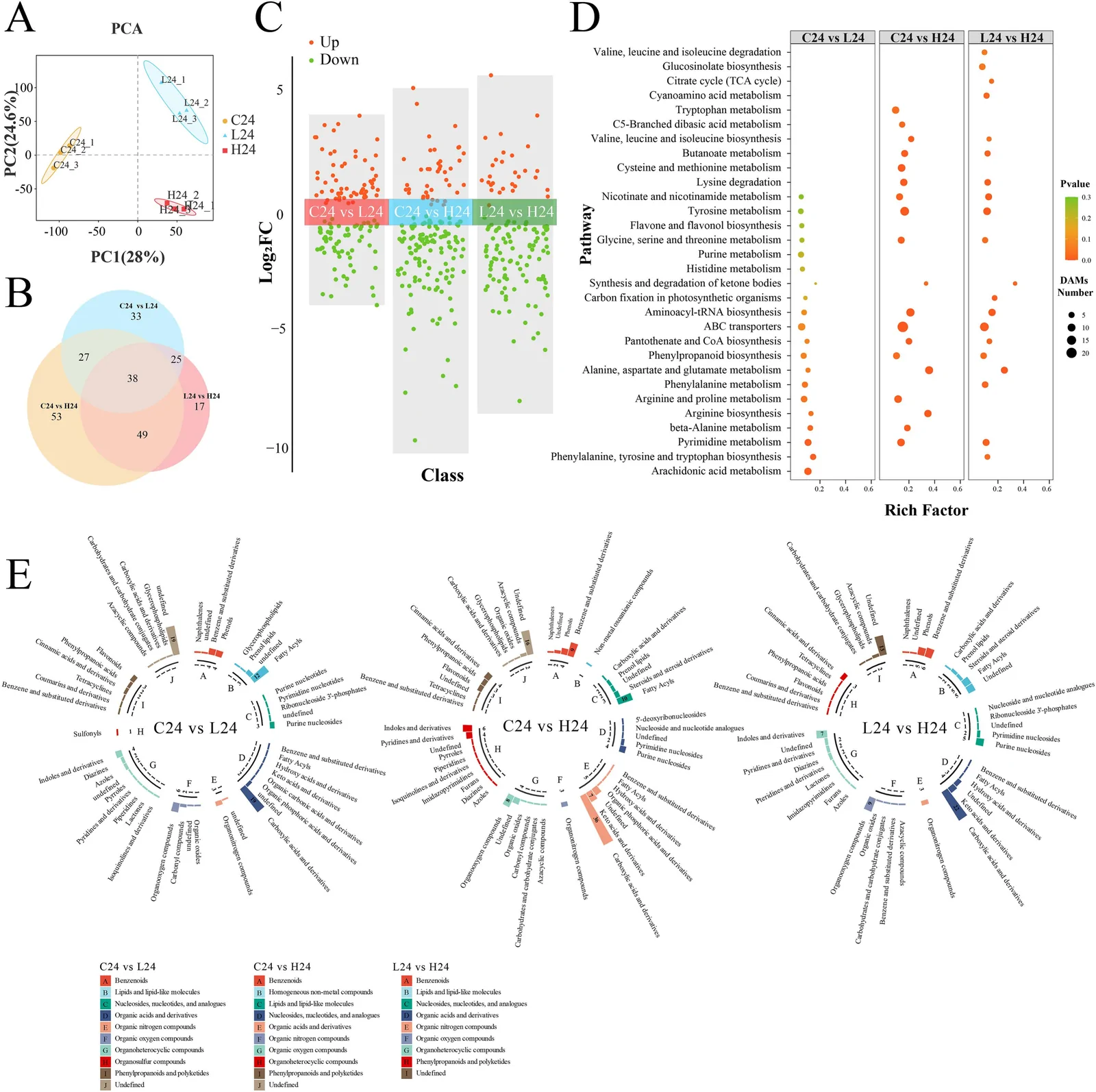
**Untargeted metabolomics analysis of *F. yunnanensis* shoots after treatment with different JA concentrations.** (A) Principal component analysis (PCA) score plot. PC1 and PC2 explained 28% and 24% of the total variation, respectively, indicating significant metabolic differences among different JA concentrations. (B) Venn diagram of differential metabolites annotated to KEGG. (C) Statistical analysis of differentially accumulated metabolites. Orange dots represented up-regulated metabolites; green dots represented down-regulated metabolites. (D) KEGG enrichment bubble plot of DAMs across three comparison groups. Bubble size represented the number of metabolites, and color intensity represented the *p*-value. (E) Classification of differential metabolites. C24, bamboo shoots treated with lanolin (vehicle control) for 24 h; L24, bamboo shoots treated with low concentration of JA (10 μmol·L^−1^) for 24 h; H24, bamboo shoots treated with high concentration of JA (150 μmol·L^−1^) for 24 h. (For interpretation of the references to color in this figure legend, the reader is referred to the web version of this article.)

Based on the differential abundance score (DA-Score) of pathways and KEGG enrichment analysis, The enriched pathways were assigned mainly to amino acid metabolism, biosynthesis of other secondary metabolites, carbohydrate metabolism, energy metabolism, lipid metabolism, membrane transport, metabolism of cofactors and vitamins, metabolism of other amino acids, nucleotide metabolism and translation (Fig. S14). Specifically, differential metabolites from low JA concentration treatment were primarily enriched in amino acid metabolism pathways (e.g., phenylalanine, tyrosine, and tryptophan biosynthesis; beta-alanine metabolism; and arginine biosynthesis) as well as flavone and flavonol biosynthesis. Compared with C24, low-concentration JA treatment significantly increased the total flavonoid content (Fig. 4J). This change was consistent with the enrichment of flavonoid-related pathways observed under this treatment. Following high JA concentration treatment, differential metabolites were mainly enriched in amino acid pathways such as alanine, aspartate, and glutamate metabolism, arginine biosynthesis, and tyrosine metabolism. The differential pathways between low and high JA concentration treatments were primarily enriched in amino acid metabolism (e.g., alanine, aspartate, and glutamate metabolism; tyrosine metabolism; and lysine degradation) and the TCA cycle (Fig. 5D).

Classification of differential metabolites revealed significant alterations in carbohydrates, amino acids, and phenolic compounds in *F. yunnanensis* shoots following JA application (Fig. 5E; Tables S9–S11). Specifically, compared with the control (24 h), the low JA concentration treatment resulted in significant changes in 9 metabolites within the carbohydrate-related categories (organic oxygen compounds, carbohydrates and carbohydrate conjugates), 24 differential metabolites within amino acid metabolism categories (carboxylic acids and derivatives, organic nitrogen compounds), and 20 metabolites within phenolic compound-related categories (benzenoids, phenylpropanoids and polyketides). The high JA concentration treatment further broadened the spectrum of differential metabolites, yielding significant changes in 8 carbohydrate-related metabolites, 40 amino acid-related metabolites, and 29 phenolic compound-related metabolites. Notably, the differential metabolites between the low and high JA concentration treatments were also predominantly found in carbohydrates, amino acids, and phenolic compounds. Accordingly, we further measured the contents of carbohydrates, free amino acids, and phenolic compounds in bamboo shoots after 24 h of JA pretreatment by using the targeted metabolomic analysis, so as to investigate the effect of JA on metabolite accumulation in *F. yunnanensis* shoots.

**Fig. 6:**
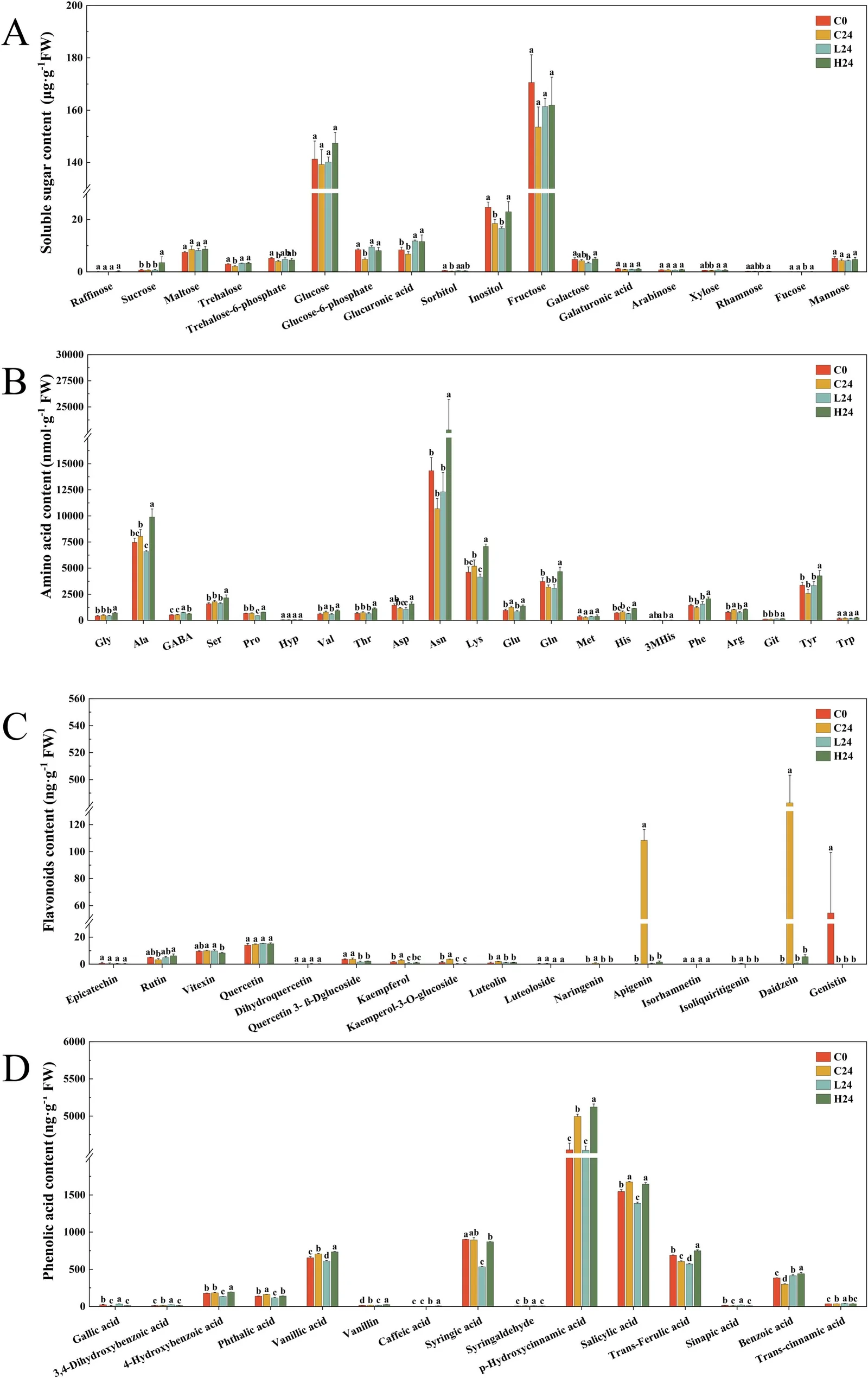
**Changes in nutrient contents of *F. yunnanensis* shoots treated with different JA concentrations based on targeted metabolomics analysis.** (A) Soluble sugar content. (B) Amino acid content. (C) Flavonoid content. (D) Phenolic acid content. Data are presented as mean ± SD from three biological replicates (*n* = 3). Statistical significance was assessed using one-way ANOVA followed by Duncan's multiple comparison test. Different lowercase letters indicated significant differences (*p* < 0.05) among treatments. C0, untreated bamboo shoots (0 h); C24, bamboo shoots treated with lanolin (vehicle control) for 24 h; L24, bamboo shoots treated with low concentration of JA (10 μmol·L^−1^) for 24 h; H24, bamboo shoots treated with high concentration of JA (150 μmol·L^−1^) for 24 h.

#### 3.4.4 Targeted metabolomic analysis of sugars in *F. yunnanensis* shoots after preharvest JA treatment

A total of 18 soluble sugars were detected in untreated *F. yunnanensis* shoots, with fructose, glucose, inositol, and maltose being the most abundant (Fig. 6A). Preharvest JA treatment did not alter the overall soluble sugar composition but significantly affected the levels of most individual soluble sugars (Fig. 6A; Fig. S8).

Except for maltose, the soluble sugar contents in the lanolin vehicle control at 24 h showed a decreasing trend compared with the untreated control at 0 h (Fig. S15A). Compared with the control (24 h), the low JA concentration treatment increased the contents of raffinose, sucrose, trehalose, trehalose-6-phosphate, glucose, glucose-6-phosphate, glucuronic acid, sorbitol, fructose, galacturonic acid, and xylose, with significant increases observed in raffinose, trehalose, glucose-6-phosphate, glucuronic acid, and xylose (Fig. S15B, C, E-G, I-J, L, N, Q). Meanwhile, low JA concentration treatment decreased the contents of maltose, inositol, galactose, arabinose, mannose, rhamnose, and fucose (Fig. S15D, K, M, O-P, R-S). In contrast, the high JA concentration treatment elevated the levels of most soluble sugars in *F. yunnanensis* shoots (Fig. S15A), particularly sucrose, trehalose, glucose-6-phosphate, glucuronic acid, and xylose, suggesting that high JA concentration promoted the soluble sugar accumulation.

#### 3.4.5 Targeted metabolomic analysis of free amino acids in *F. yunnanensis* shoots after preharvest JA treatment

The targeted amino acid metabolomics revealed 21 amino acids in control shoots at both 0 h and 24 h, among which Asp, Ala, Lys, and Gln were the most abundant (Fig. 6B). The preharvest JA treatment did not alter the amino acid composition of *F. yunnanensis* shoots but significantly affected the contents of various amino acids, with high JA concentration exerting a stronger effect (Fig. 6B; Fig. S16A). During normal shoot growth (0 h to 24 h), the contents of most amino acids, including Gly, Ala, Ser, and Val, increased. In contrast, those of Asp, Asn, and Gln decreased (Fig. 6B). Compared with the control (24 h), the low JA concentration treatment increased the levels of GABA, Hyp, Cit, Met, Phe, and Tyr, but decreased the levels of Ala, Asp, Glu, Gln, and Lys in *F. yunnanensis* shoots (Fig. S16C, D, G, J, L-O, R, T, U). By contrast, the high JA concentration treatment significantly enhanced the accumulation of most amino acids in *F. yunnanensis* shoots (Fig. 6B; Fig. S16A). Except for GABA and Hyp, the remaining amino acids were all significantly more abundant than in the control (24 h), indicating that high concentration of JA altered the levels of most amino acids in bamboo shoots.

#### 3.4.6 Targeted metabolomic analysis of phenolic compounds in *F. yunnanensis* shoots after preharvest JA treatment

Targeted metabolomics revealed 16 flavonoids across all treatment groups in *F. yunnanensis* shoots (Fig. 6C), among which quercetin, vitexin, and rutin were abundant in all groups. Notably, naringenin, apigenin, daidzein, and isoliquiritigenin were detected exclusively in control shoots at 24 h, isorhamnetin was found only in the low JA concentration-treated shoots, and genistin was present only in the control (0 h) (Fig. 6C). Compared with the control (0 h), the lanolin vehicle control at 24 h showed increased contents of vitexin, quercetin, kaempferol, kaempferol-3-O-glucoside, luteolin, and luteoloside, particularly kaempferol, kaempferol-3-O-glucoside, and luteolin (Fig. S17A, D–K), whereas epicatechin, rutin, dihydroquercetin, and quercetin-3-O-glucoside decreased slightly (Fig. S17B, C, F, G). The effects of JA pretreatment on flavonoid accumulation were concentration-dependent. Under low JA concentration treatment, rutin, quercetin, and dihydroquercetin were slightly more abundant than in the lanolin vehicle control at 24 h, whereas epicatechin, vitexin, quercetin-3-O-glucoside, kaempferol, kaempferol-3-O-glucoside, luteolin, and luteoloside decreased in content (Fig. S17C–K). Compared with the lanolin vehicle control at 24 h, the high JA concentration treatment decreased the levels of most flavonoids but significantly promoted rutin accumulation (Fig. S17A, C). Furthermore, relative to the low JA concentration treatment, the high JA concentration treatment enhanced the accumulation of rutin, quercetin-3-O-glucoside, kaempferol, and luteolin in bamboo shoots.

A total of 15 phenolic acids were detected in bamboo shoots, with p-hydroxycinnamic acid, salicylic acid, syringic acid, trans-ferulic acid, and vanillic acid being the most abundant (Fig. 6D). The preharvest JA treatment did not alter the phenolic acid profile but increased the accumulation of most phenolic acids in *F. yunnanensis* shoots (Fig. 6D; Fig. S18A). Most phenolic compounds increased during bamboo shoot aging, with particularly pronounced increases in 3,4-dihydroxybenzoic acid, phthalic acid, vanillic acid, vanillin, syringaldehyde, p-hydroxycinnamic acid, salicylic acid, and trans-cinnamic acid. Compared with normally grown shoots, the low JA concentration treatment significantly altered phenolic compound contents, with the contents of gallic acid, 3,4-dihydroxybenzoic acid, caffeic acid, syringaldehyde, sinapic acid, benzoic acid, and trans-cinnamic acid increasing significantly, whereas the contents of 8 phenolic compounds, such as syringic acid, vanillic acid, p-hydroxycinnamic acid, and trans-ferulic acid, decreased significantly (Fig. 6D; Fig. S18). In contrast, the high JA concentration treatment slightly increased gallic acid content and significantly increased the contents of 4-hydroxybenzoic acid, vanillic acid, vanillin, caffeic acid, p-hydroxycinnamic acid, trans-ferulic acid, and benzoic acid, while reduced the levels of 3,4-dihydroxybenzoic acid, phthalic acid, syringic acid, syringaldehyde, sinapic acid, and trans-cinnamic acid in *F. yunnanensis* shoots.

Gossypol was also detected in *F. yunnanensis* shoots. Its content increased significantly during normal growth. However, treatment with different concentrations of exogenous JA significantly reduced gossypol content compared with the 24 h control group (Fig. S19).

### 3.5 Correlation analysis between nutritional components of *F. yunnanensis* shoots and preharvest JA treatment concentration

Correlation analysis revealed that various nutrient contents in *F. yunnanensis* shoots were closely correlated with the preharvest JA concentrations (Fig. 7). Specifically, the contents of various phenolic compounds, amino acids, and carbohydrates showed positive associations with the JA treatment level, including caffeic acid, trans-ferulic acid, vanillin, Asn, starch, Gln, Gly, Thr, His, Lys, Phe, Cit, Asp, Ala, Tyr, Ser, inositol, sucrose, and glucose. Further analysis revealed that the contents of caffeic acid, trans-ferulic acid, vanillin, starch, and various amino acids (including Asn and Gln) were significantly positively correlated with JA concentration, suggesting that the JA application might promoted their accumulation. In contrast, nutrients negatively correlated with JA concentration mainly included syringaldehyde, vitexin, and some flavonoids. The content of syringaldehyde was highly significantly negatively correlated with JA concentration, while that of vitexin was significantly negatively correlated, suggesting that the JA application reduced their accumulation. Overall, the preharvest JA treatment was associated with changes in the contents of several nutrients in *F. yunnanensis* shoots. As JA concentration increased, various phenolic acids, amino acids, and soluble sugars accumulated, whereas some flavonoids decreased.

**Fig. 7:**
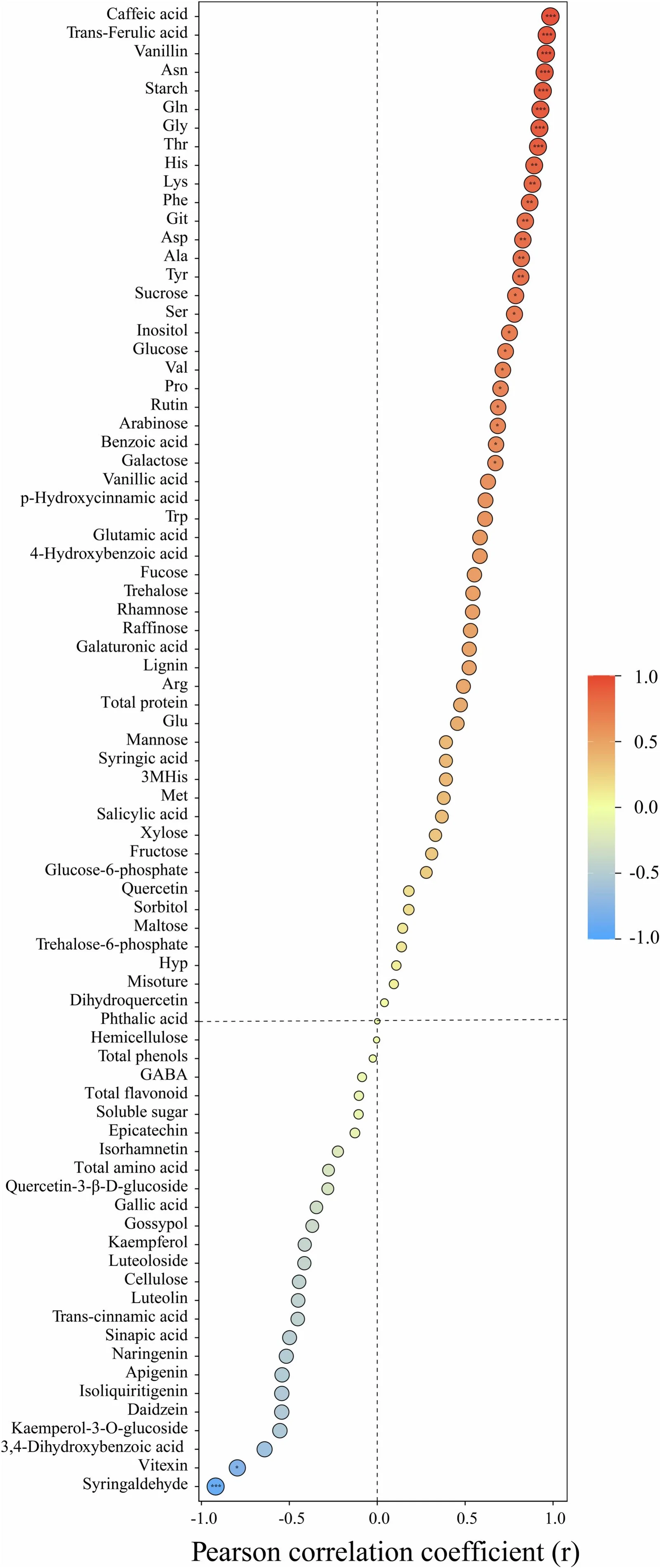
**Correlation analysis between JA concentration and nutrient contents.** Pearson correlation analysis was performed using nine biological samples from the C24, L24, and H24 groups. Color intensity indicated the strength of the correlation. Red circles represented positive correlation; blue circles represented negative correlation. * *p* < 0.05; ** *p* < 0.01; *** *p* < 0.001. (For interpretation of the references to color in this figure legend, the reader is referred to the web version of this article.)

Bamboo shoot cell wall composition and content significantly affect taste. Among the cell wall components in *F. yunnanensis* shoots, the lignin content showed a positive association with the JA concentration, whereas the cellulose content showed a negative association and the hemicellulose content showed a weak negative association. This indicated that the preharvest JA treatment did not promote simultaneous accumulation of all cell wall components but rather induced lignin deposition. Since lignin accumulation was closely associated with tissue hardening, the preharvest JA treatment might promote shoot lignification and compromise taste. These results suggested that JA treatment was associated with differential changes in cell wall composition, with lignin showing an increasing trend and cellulose and hemicellulose showing decreasing or weakly decreasing trends across the tested treatment levels. However, the correlation analysis did not demonstrate that JA directly induced lignin deposition. Because lignin accumulation is associated with tissue hardening, this pattern might indicate a potential influence of JA treatment on shoot texture, which requires further physiological validation.

## 4 Discussion

Bamboo shoots are rich in nutrients, including primary and secondary metabolites such as carbohydrates, proteins, amino acids, and phenolic compounds, particularly flavonoids and phenolic acids (Ma et al., 2024). In recent years, studies on bamboo shoots have mainly focused on their rapid growth and associated changes in anatomical structure and sugar metabolism (Wei et al., 2017; Wang, Pei, et al., 2020), as well as postharvest preservation (Yu et al., 2021). However, studies on metabolite changes across different growth stages and on the effects of preharvest JA treatment on metabolite accumulation remain limited. In the present study, we systematically analyzed nutritional changes in *F. yunnanensis* shoots across different growth stages and investigated the effects of preharvest JA treatment at different concentrations on metabolite accumulation.

### 4.1 Differences in nutritional and metabolic composition across developmental stages

The composition and content of cell wall components in vegetables directly affect their palatability and quality (Wang et al., 2023). Cellulose, hemicellulose and lignin are important structural components of bamboo shoot cell walls. An increase in lignin content might lead to a higher degree of lignification, thereby increasing the roughness and hardness of bamboo shoots and reducing their quality and edibility (Wang et al., 2023). In this study, the contents of cellulose, hemicellulose and lignin in the bamboo shoots of *F. yunnanensis* showed an increasing trend with height growth, which led to the continuous increase of the aging degree of bamboo shoots. This pattern was similar to the results of Huang et al. (2025) and Yang, Li, et al. (2021). It is worth noting that the levels of cellulose, hemicellulose, and lignin at the ES stage remained low because of the high moisture content. Nevertheless, bamboo shoots at this stage still serve as a good source of dietary fiber. As shoot development progressed, cell wall components continued to accumulate, potentially making their tissues harder and coarser and adversely affecting their texture and palatability.

The nutritional composition of *F. yunnanensis* shoots also changes during development. With the aging of bamboo shoots, the content of most nutrients decreased to varying extents, which was consistent with the results of Nirmala et al. (2007). Carbohydrates are important determinants of the nutritional value, palatability, and postharvest physiological properties of bamboo shoots. In the present study, starch, soluble sugar, and major soluble sugars, including raffinose, sucrose, fructose, and glucose, were more abundant at the Sb stage than at the ES and AS stages, which might favor subsequent flavor formation. During the development of *F. yunnanensis*, the contents of most soluble sugars initially decreased and then increased, remaining relatively low during the ES stage. This pattern may be associated with carbohydrate utilization during rapid shoot growth and changes in tissue moisture content, although the underlying mechanisms require further investigation. By contrast, glucose-6-phosphate, trehalose-6-phosphate and sugar alcohols (such as sorbitol and inositol) showed slight accumulation at the ES stage, which might be related to the active metabolic changes during rapid shoot growth. It is worth noting that sugar derivatives such as glucuronic acid, xylose, and galacturonic acid were closely related to cell wall components and texture characteristics (Schmitz et al., 2019; Yang, Yi, et al., 2021; Paz-Cedeno et al., 2022). Their changes across developmental stages may reflect differences in cell wall-related metabolism. However, their relationships with tissue texture require further validation through textural measurements and sensory evaluation. In the present study, as bamboo shoots entered the aged stage, the soluble sugars such as raffinose, glucose, and fructose increased again. This increase could be attributed to starch degradation and a decrease in nutrient consumption following shoot aging. In addition, glucuronic acid, xylose, and galacturonic acid, which serve as precursors for cell wall formation, decreased during bamboo shoot aging. This trend was consistent with the continuous accumulation of cellulose and lignin, suggesting an association between carbohydrate contents and cell wall-related changes.

Bamboo shoots contain a variety of amino acids required by the human body and may serve as a dietary source of amino acids (Ma et al., 2024). In this study, 20 amino acids were identified in *F. yunnanensis* shoot buds and shoots. Among them, lysine, phenylalanine, threonine, tryptophan, valine, and histidine are essential amino acids for humans, indicating that *F. yunnanensis* shoots have nutritional value as an amino acid source. Their amino acid composition also differed from that of other bamboo species. Asp was the predominant amino acid in *Dendrocalamus brandisii* and *Phyllostachys pubescens* shoots, whereas Pro was most abundant in *Dendrocalamus latiflorus* shoots (Hu et al., 2000; Liu et al., 2021; Shi et al., 2025). In addition, the amino acid composition changed across developmental stages. Except for Cit and Glu, most amino acids were more abundant at the Sb stage than at the ES and AS stages, possibly reflecting greater accumulation of nutrients and metabolic substrates in shoot buds for subsequent growth. Notably, Cit levels peaked at the ES stage, which represents the edible stage. Given that Cit is involved in ammonia detoxification and serves as a key precursor for nitric oxide (NO) production (Fish & Bruton, 2014), its elevated accumulation at this stage might enhance the edible value of bamboo shoots. Beyond their nutritional role, free amino acids are important determinants of bamboo shoot flavor, with their composition and relative abundance jointly shaping umami, sweetness, and bitterness (Ma et al., 2024). *F. yunnanensis* shoots contained relatively high levels of umami- and sweet-taste-associated amino acids, whereas some bitter-taste-associated amino acids were present at relatively low levels. This suggests that *F. yunnanensis* shoots may have a characteristic amino acid profile related to flavor, although their actual sensory properties require further validation through sensory evaluation.

Phenolic compounds are important secondary metabolites in bamboo shoots, mainly comprising flavonoids and phenolic acids. Some of these compounds can scavenge free radicals, inhibit reactive oxygen species (ROS) production, and suppress the activites of related oxidative enzymes (Ma et al., 2024; Wang, Chen, et al., 2020). Flavonoids are also considered to have considerable potential for the prevention and treatment of arteriosclerosis, cancer, diabetes, and neurodegenerative diseases (Wang, Chen, et al., 2020). The enrichment of flavonoid-related pathways in the untargeted metabolomics analysis, together with differences in total flavonoid content across developmental stages, suggests that flavonoid-related metabolism changes during bamboo shoot development. The relatively high flavonoid levels at the Sb stage may have implications for phenolic composition; antioxidant and nutritional effects were not directly assessed. Notably, quercetin, vitexin, and rutin predominated at the Sb stage. These compounds have been widely reported to possess antioxidant, anti-inflammatory, and cardioprotective activities (Al-Khayri et al., 2022), which might enhance the edible value of *F. yunnanensis* shoots at this stage.

Similarly, 15 phenolic acids were identified in *F. yunnanensis* shoots, with p-hydroxycinnamic acids, vanillic acid, trans-ferulic acid, and syringic acid being the major compounds. Phenolic acids are generally classified into hydroxybenzoic acids and hydroxycinnamic acids, the latter exhibiting higher antioxidant activity (Lourenço Neto et al., 2018). Park and Jhon (2010) identified 8 phenolic acids in *P. pubescens* and *Phyllostachys nigra* shoots, among which hydroxybenzoic acid, dihydroxybenzoic acid, and syringic acid were the predominant compounds. By contrast, p-hydroxycinnamic acid was abundant across all developmental stages of *F. yunnanensis* shoots, potentially enhancing antioxidant activity and edible value. In addition to their potential antioxidant activity, phenolic compounds may contribute to the sensory attributes and overall quality of fruits and vegetables (Pott et al., 2019). Although high levels of catechin, epicatechin, rutin, and caffeic acid enhance reducing power and radical-scavenging activity, they can negatively affect palatability (Lesschaeve & Noble, 2005; Ma et al., 2024). In this study, shoot buds of *F. yunnanensis* contained relatively high levels of catechin, epicatechin, rutin, and caffeic acid, indicating developmental stage-dependent differences in the accumulation of these phenolic compounds. As some phenolic compounds may contribute to bitterness or astringency (Kalinová et al., 2018), changes in their levels may affect the sensory properties of shoot buds. However, the actual contributions of phenolic compounds to bitterness and astringency depend on factors such as compound concentration, matrix composition, and interactions among compounds, and therefore require further validation through sensory evaluation.

### 4.2 Effects of preharvest JA treatment on metabolite contents

Preharvest treatment is an important approach for improving postharvest quality in fruits and vegetables, as it can optimize quality before harvest and thereby influence subsequent changes during storage (Zhao et al., 2024). Among various preharvest strategies, exogenous plant hormone application has received considerable attention for its ability to regulate quality-related metabolism. In recent years, JA and its derivative MeJA have attracted increasing attention for their potential to regulate fruit and vegetable quality. Exogenous JAs can alter the levels of major metabolites, including sugars, amino acids, and phenolic compounds, thereby affecting nutritional value and quality (Wang & Zheng, 2005; Kucuker et al., 2014; Zhang et al., 2024; Baek et al., 2021; Wang, Mao, et al., 2022). However, their effects are not always positive, varying with crop species and application concentration. Tan et al. (2018) found that exogenous MeJA accelerated leaf senescence and increased endogenous JA levels in *Brassica rapa*. Fang et al. (2020) reported that exogenous MeJA accelerated chlorophyll degradation and induced yellowing in *Brassica oleracea*. Ji et al. (2016) further showed that high JA concentrations inhibited plant growth. Our results showed that preharvest treatment with low-concentration JA promoted bamboo shoot growth and was accompanied by reduced moisture content. By contrast, the high JA concentration treatment delayed shoot growth and increased moisture accumulation, suggesting that preharvest JA treatment might help retard bamboo shoot growth.

JA has been proven to regulate plant cell wall metabolism and lignin accumulation (Mielke & Gasperini, 2019). This study found that low JA concentration treatment increased cellulose, hemicellulose, and lignin contents in bamboo shoots, whereas high JA concentration treatment slightly reduced cellulose content while significantly increasing hemicellulose and lignin. This finding differed from those of Ding et al. (2024), who reported that MeJA reduced cellulose and hemicellulose contents during apple storage. Regarding lignin content, our results were consistent with those of Concha et al. (2013), who found that MeJA treatment promoted lignin accumulation in *Fragaria chiloensis* fruit during storage. Notably, cellulose, hemicellulose, and lignin form the main structural framework of plant cell walls, and changes in their contents can affect vegetable texture. The significant increase in lignin content following high-concentration JA treatment suggests that JA may influence the formation and accumulation of cell wall components in bamboo shoots. However, as the activities of key lignin biosynthetic enzymes and the expression of related genes were not measured in this study, the underlying regulatory mechanisms cannot be clearly elucidated based on the metabolomic data alone.

Carbohydrates are important determinants of flavor and quality in bamboo shoots (Huang et al., 2025). In the present study, the preharvest exogenous JA treatment significantly affected carbohydrate metabolism and accumulation in *F. yunnanensis* shoots, exhibiting a clear concentration-dependent effect. The low JA concentration treatment promoted the accumulation of soluble sugar and xylose, while reducing starch content. By contrast, the high JA concentration treatment promoted the accumulation of most sugars, including sucrose, fructose, and glucose. This finding was consistent with those of Wang, Zhang, Lv, et al. (2022), who reported that exogenous MeJA increased soluble sugars, proteins, sulfur compounds, and aromatic volatiles in hydroponically grown *Allium tuberosum*, thereby improving its characteristic flavor. Kucuker et al. (2014) also reported that the preharvest MeJA promoted carbohydrate synthesis in *Prunus salicina*, maintaining flesh firmness, increasing phenolic content, and enhancing antioxidant activity during ripening. In contrast to findings from most studies, Tytgat et al. (2013) reported that JA treatment reduced sugar concentrations in *B. oleracea*, indicating that exogenous jasmonate treatment does not consistently promote sugar accumulation. These differences may be related to plant species, tissue developmental stage, and hormone concentration. *F. yunnanensis* shoots are in a phase of rapid elongation, during which sugars provide energy for growth and serve as substrates for cell wall formation and other metabolic processes. Compared with C0, most soluble sugars decreased in C24, whereas sucrose, fructose, glucose, and other sugars were present at higher levels following high-concentration JA treatment than in C24. The inhibition of shoot elongation by high-concentration JA, accompanied by increased levels of some sugars, may reflect reduced sugar utilization for growth.

Proteins and amino acids are another class of JA-regulated metabolites, and exogenous JA pretreatment can significantly increase total soluble protein and Pro contents in plants (Wang et al., 2023). However, in the present study, the low JA concentration treatment significantly reduced protein content, whereas the high concentration of JA induced only a slight increase. This discrepancy may be related to JA concentration. *F. yunnanensis* shoots are in a phase of rapid elongation, and low-concentration JA promoted shoot growth. Protein synthesis and turnover are closely associated with growth requirements. However, as protein synthesis- and degradation-related indicators were not measured in this study, the specific causes of the changes in protein content remain unclear. By contrast, the high JA concentration treatment markedly altered amino acid abundance profiles and showed a stronger promotive effect on amino acid accumulation. Gly, Ala, Ser, and Val showed an increasing trend in C24 compared with C0 and were further significantly elevated following high-concentration JA treatment compared with C24. This suggests that their accumulation exceeded the changes observed during normal shoot elongation. This may be associated with the inhibition of shoot elongation by high-concentration JA, potentially reducing amino acid utilization for growth. In our study, the increases in some essential amino acids and sweet- and umami-taste-associated amino acids indicate that JA treatment altered the nutritional and flavor-related composition of bamboo shoots. However, its effects on nutritional contribution and sensory quality require further evaluation. Amino acid responses to jasmonates also vary among studies. Tytgat et al. (2013) reported that JA treatment reduced amino acid levels in *B. oleracea*, consistent with the decreases in some free amino acids observed following low-concentration JA treatment in the present study. However, Wang, Zhang, Xie, et al. (2022) found that the preharvest treatment with 500 and 800 μmol·L^−1^ MeJA significantly increased Lys, Ile, Val, Met, Gly, Asp, Pro, and Glu levels in hydroponically grown *A. tuberosum*. These differences may be related to plant species, the treatment compound (JA or MeJA), treatment concentration, application method, and experimental conditions. *F. yunnanensis* shoots are young organs undergoing rapid elongation, and their amino acid supply is closely associated with growth requirements. Their metabolic status may differ from that of other plant materials, such as Chinese chive leaves, which may partly explain the different responses observed.

Phenolic compounds are major secondary metabolites in fruits and vegetables, crucial for scavenging reactive oxygen species, enhancing antioxidant capacity, and promoting human health (Ma et al., 2024). Numerous studies have shown that JA, when applied as a preharvest treatment, acts as a key signaling molecule in regulating the accumulation of these compounds. Preharvest MeJA treatment has been shown to significantly increase total flavonoids in florets of *B. oleracea* and *B. rapa* (Baek et al., 2021; Wang, Mao, Liang, et al., 2022), elevate kaempferol and rutin in *P. salicina* (Kucuker et al., 2014), and enhance quercetin and kaempferol in *Rubus* sp. and *R. idaeus* (Wang et al., 2008; Wang & Zheng, 2005). In our study, preharvest exogenous JA treatment generally reduced most flavonoid levels in *F. yunnanensis* shoots, but JA concentration exerted differential effects. Compared with C24, total flavonoid content increased following both JA treatments, while untargeted metabolomics revealed the enrichment of differential metabolites in flavonoid-related pathways. Together, these findings suggest that JA treatment was associated with changes in flavonoid-related metabolism, although individual flavonoid compounds did not necessarily show a consistent increase. A low JA concentration promoted the accumulation of total flavonoids, quercetin, isorhamnetin, and dihydroquercetin, whereas a high concentration significantly increased rutin content, a finding consistent with Wang, Zhang, Xie, et al. (2022). Rutin is the predominant flavonoid in these shoots. The increase in rutin represented a compound-specific response to JA treatment.

Exogenous JAs have also been shown to promote phenolic acid accumulation in plants, thereby enhancing antioxidant capacity (Martínez-Esplá et al., 2017; Złotek et al., 2019). During normal shoot elongation, the levels of vanillic acid, vanillin, and p-hydroxycinnamic acid increased and were further elevated following high-concentration JA treatment compared with C24, suggesting that JA may promote the accumulation of certain phenolic acids.Previous studies have also shown that exogenous JA treatment increases phenolic acid levels and antioxidant activity in various fruits and vegetables. Złotek et al. (2019) reported that exogenous JA induced phenolic accumulation in *Triticum aestivum*, thereby enhancing its antioxidant and anti-inflammatory activities. Martínez-Esplá et al. (2017) demonstrated that preharvest MeJA treatment increases phenolic compound content in *C. cardunculus*. Wang, Zhang, Xie, et al. (2022) further observed similar effects in *A. tuberosum*, and Zhang et al. (2024) also reported the same for *V. vinifera*. In addition, preharvest MeJA treatment increased phenolic acid accumulation in *P. salicina*, including caffeic acid, p-coumaric acid, and ferulic acid (Kucuker et al., 2014). This suggests that appropriate preharvest JA treatment may improve the quality of such fruits and vegetables. In this study, the preharvest JA treatment exhibited a concentration-dependent effect on phenolic acid metabolism in *F. yunnanensis* shoots. The low JA concentration treatment promoted shoot growth but decreased most phenolic acids, with particularly pronounced decreases in p-hydroxycinnamic acid, salicylic acid, and syringic acid. In contrast, the high JA concentration treatment increased the contents of total phenolics and most phenolic acids. These results are consistent with the findings in *A. tuberosum* (Wang, Zhang, Xie, et al., 2022), *L. sativa* (Almeida et al., 2025), and *B. rapa* (Baek et al., 2021). Notably, caffeic acid-, p-coumaric acid-, and ferulic acid-related compounds are associated with phenylpropanoid metabolism. Therefore, changes in these metabolites, together with changes in lignin content, suggest that JA treatment may affect phenolic metabolism and cell wall-related processes in *F. yunnanensis* shoots. However, the accumulation of these phenolic acids does not directly demonstrate their increased utilization as precursors for lignin biosynthesis, as lignin monomer composition, related enzyme activities, gene expression, and metabolic fluxes were not assessed in this study.

In summary, low-concentration JA treatment promoted bamboo shoot growth and was accompanied by decreases in soluble sugar and protein contents, whereas high-concentration JA treatment inhibited growth and increased the contents of most soluble sugars, amino acids, and phenolic acids. However, high-concentration JA treatment also reduced most flavonoids and increased lignin content, indicating differential responses among quality-related components. The effects of JA on edible quality require further evaluation, as textural properties, sensory attributes, and shelf life were not assessed.

Several limitations should be acknowledged when interpreting the JA-related metabolic responses observed in this study. This study examined metabolic responses to JA treatment after 24 h. The lack of time-course data, enzyme activity measurements, and gene expression analysis limits further interpretation of the underlying regulatory mechanisms. Future studies should combine time-course metabolomics with physiological and molecular analyses, alongside textural and sensory evaluations, to clarify the mechanisms underlying JA-induced changes and assess its potential for preharvest application.

## 5 Conclusion

This study systematically characterized changes in quality-related components across the shoot bud, edible shoot, and aged shoot stages of *F. yunnanensis* and their responses to different JA concentrations. The shoot bud stage was characterized by relatively high levels of starch, soluble sugars, and amino acids. At the edible shoot stage, most nutrition-related components decreased, while moisture content increased and cellulose and lignin contents remained relatively low. The aged shoot stage showed further accumulation of cell wall components, accompanied by changes in some phenolic and bitterness-associated compounds. JA treatment exhibited concentration-dependent effects. Low-concentration JA treatment generally reduced the contents of some soluble sugars, amino acids, and phenolic compounds. In contrast, high-concentration JA treatment increased the contents of most soluble sugars and amino acids, rutin, and major phenolic acids such as p-coumaric acid, while reducing most flavonoids and increasing lignin content. These findings highlight the differential responses of quality-related components to JA treatment and suggest that its preharvest application requires further evaluation in terms of treatment concentration, postharvest quality, shelf life, and food safety.

The following are the supplementary data related to this article.

**Fig. S1:**
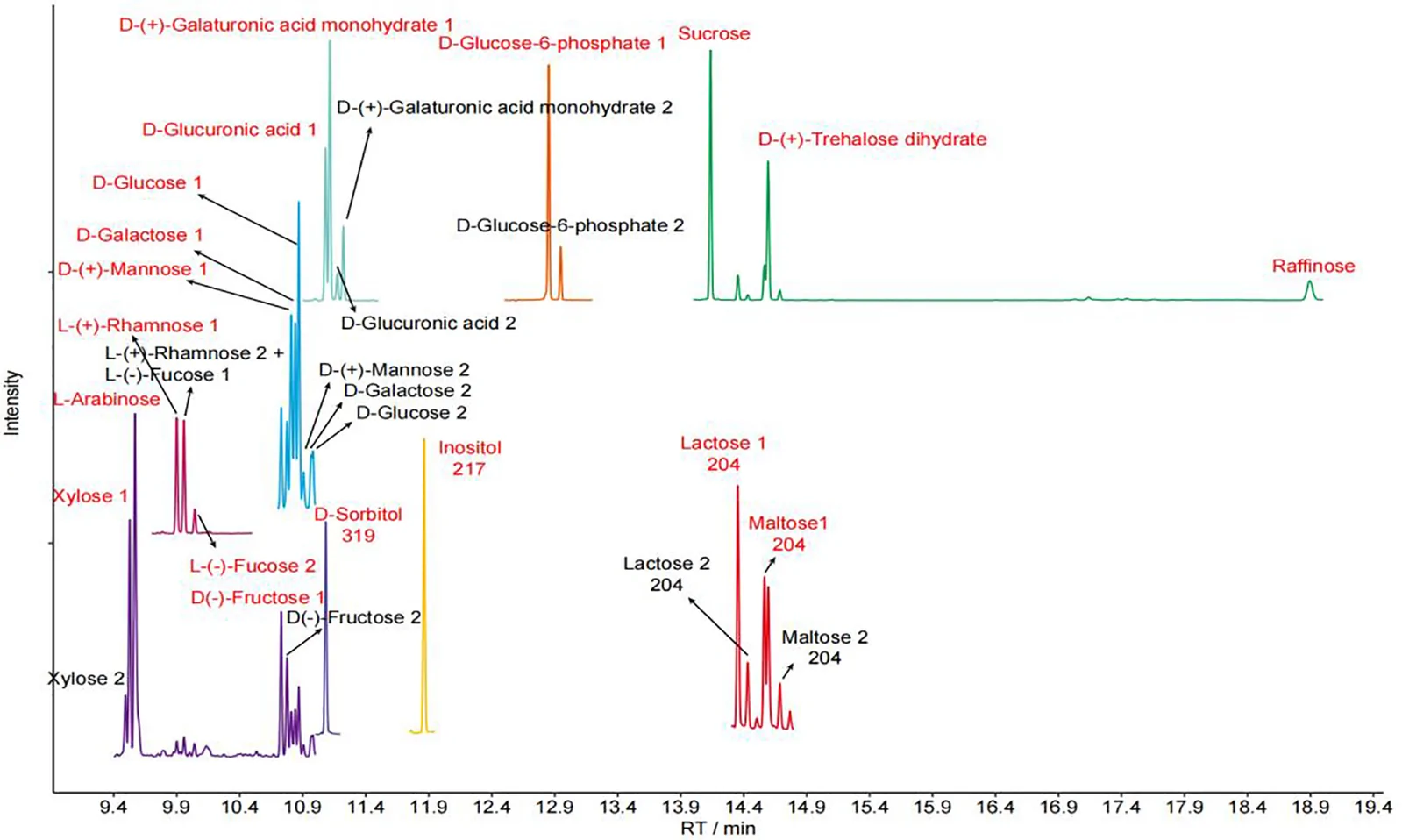
**GC–MS selected ion monitoring (SIM) chromatograms of target carbohydrate standards.** The corresponding compounds are annotated in the chromatograms.

**Fig. S2:**
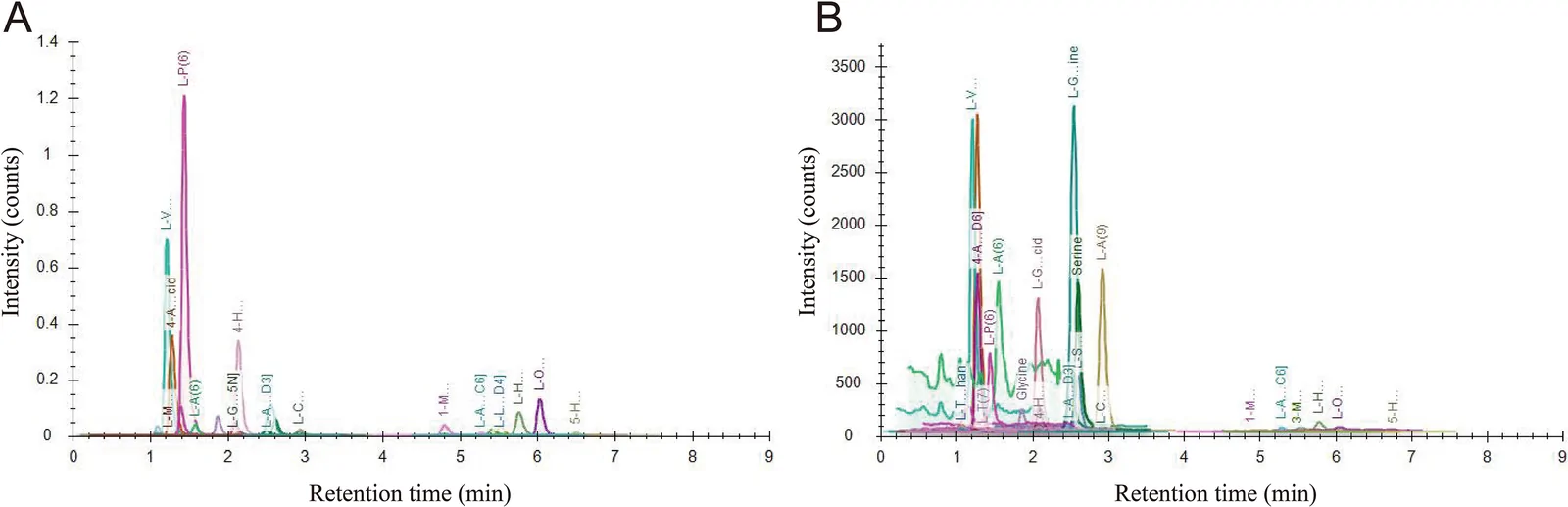
**Representative extracted ion chromatograms of selected target amino acids.** (A) standard solution; (B) bamboo shoot samples from different developmental stages and JA treatments. The corresponding compounds are annotated in the chromatograms.

**Fig. S3:**
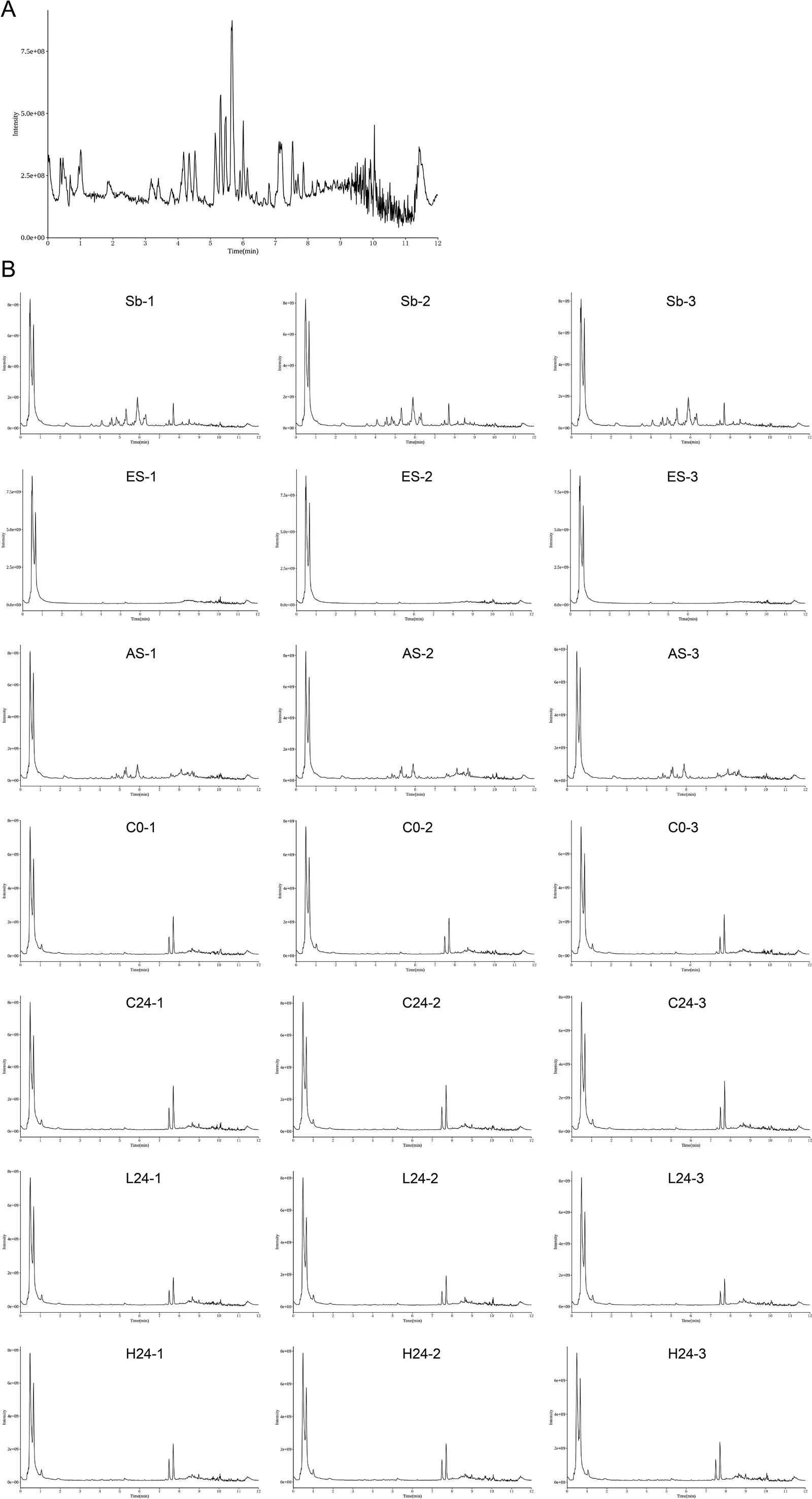
**Chromatograms from the targeted analysis of phenolic compounds.** (A) standard solution; (B) bamboo shoot samples from different developmental stages and JA treatments. Numbers 1–3 indicate biological replicates.

**Fig. S4:**
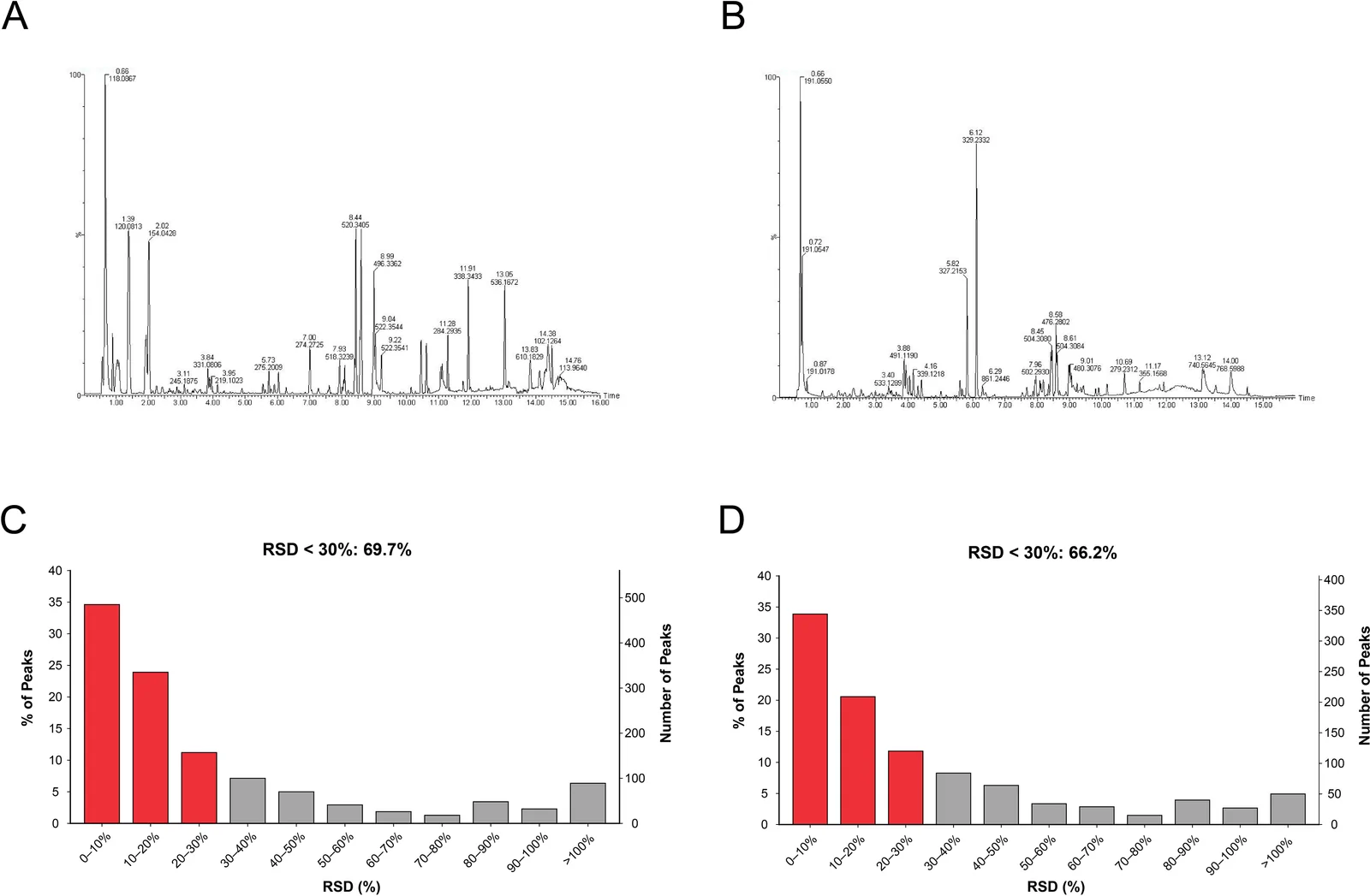
**Total ion chromatograms and relative standard deviation (RSD) distributions of QC samples for untargeted metabolomic analysis of bamboo shoots at different developmental stages.** (A, B) Total ion chromatograms in positive and negative ion modes, respectively. (C, D) RSD distributions of peak areas across QC samples in positive and negative ion modes, respectively. Red bars indicate RSD < 30%, accounting for 69.7% and 66.2% of the detected features, respectively.

**Fig. S5:**
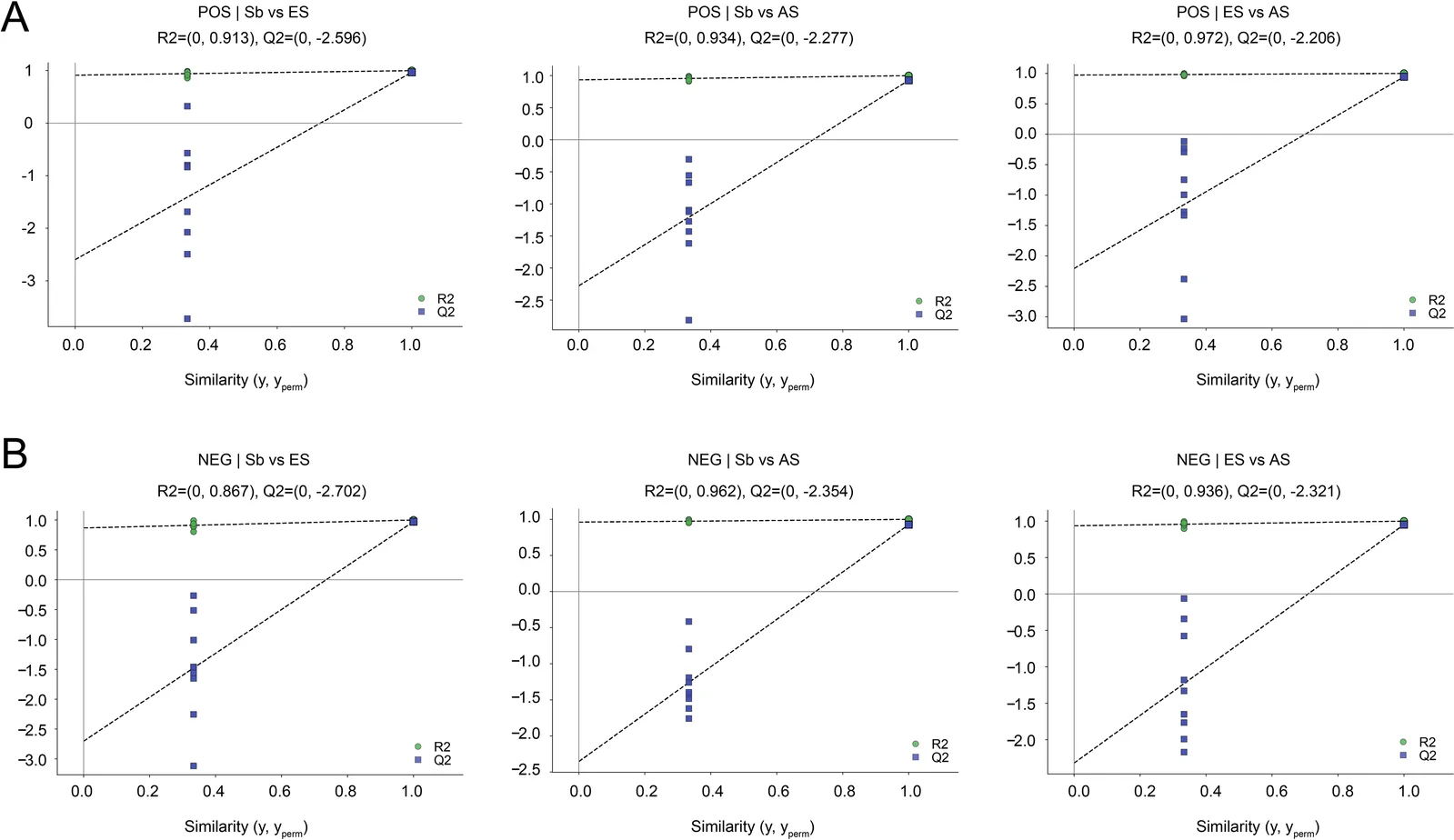
**Permutation tests of OPLS-DA models for bamboo shoots at different developmental stages.** (A) Positive ion mode. (B) Negative ion mode. Green circles and blue squares represent R^2^Y and Q^2^ values, respectively; dashed lines indicate linear regression fits.

**Fig. S6:**
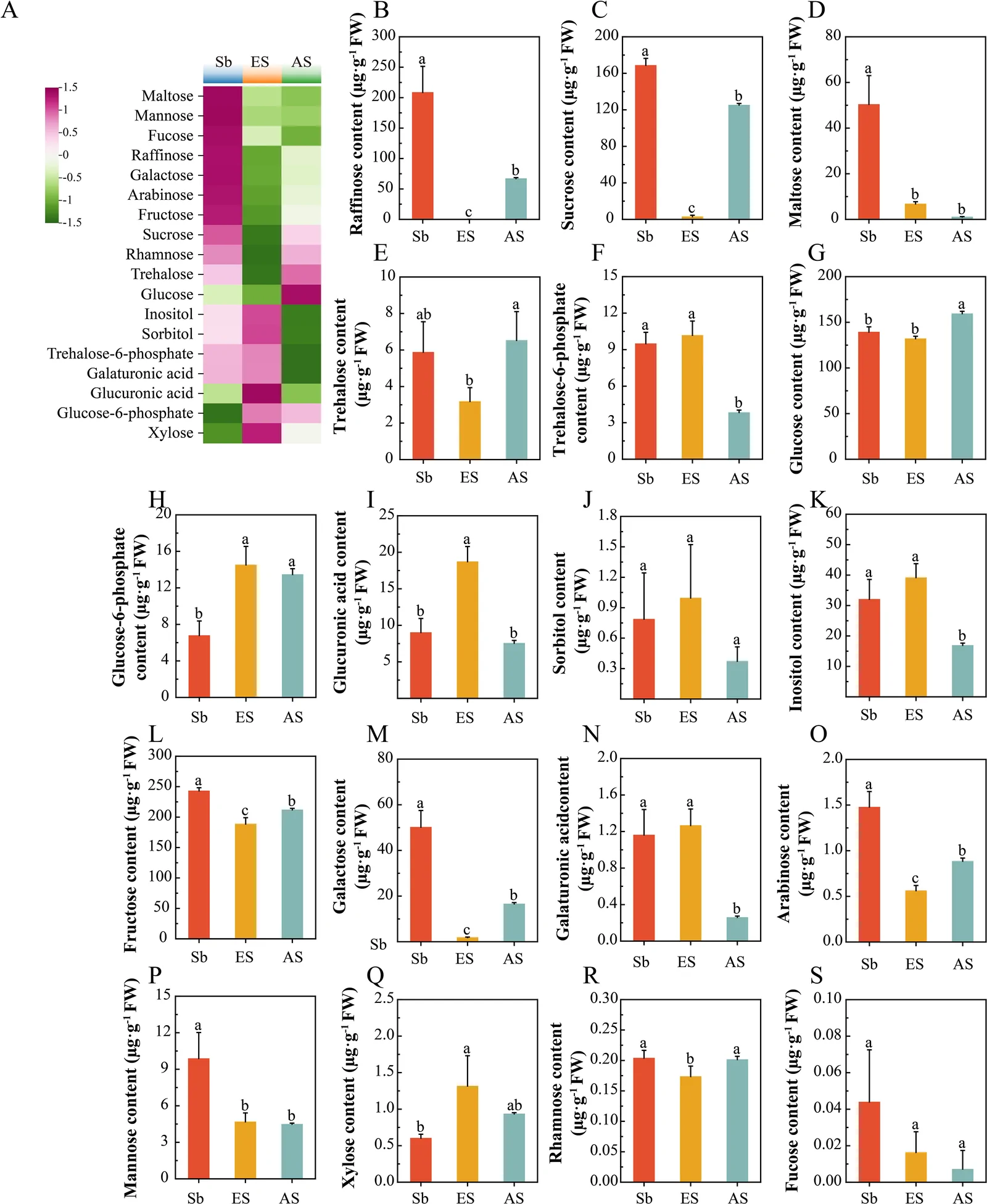
**Soluble sugar contents of *F. yunnanensis* shoots at different growth stages.** (A) Heat map showing changes in soluble sugar contents at different growth stages. Data were *Z*-score standardized. The color gradient reflected the abundance of each soluble sugar across different developmental stages. Purple-red: higher relative content; green: lower relative content. (B) Raffinose content; (C) Sucrose content; (D) Maltose content; (E) Trehalose content; (F) Trehalose-6-phosphate content; (G) Glucose content; (H) Glucose-6-phosphate content; (I) Glucuronic acid content; (J) Sorbitol content; (K) Inositol content; (L) Fructose content; (M) Galactose content; (N) Galacturonic acid content; (O) Arabinose content; (P) Mannose content; (Q) Xylose content; (R) Rhamnose content; (S) Fucose content. Data are presented as mean ± SD from three biological replicates (*n* = 3). Statistical significance was assessed using one-way ANOVA followed by Duncan's multiple comparison test. Different lowercase letters indicated significant differences (*p* < 0.05) among growth stages. Sb, shoot buds; ES, normal edible bamboo shoots (30–50 cm in height); AS, aged bamboo shoots, whose first internode had completed elongation growth.

**Fig. S7:**
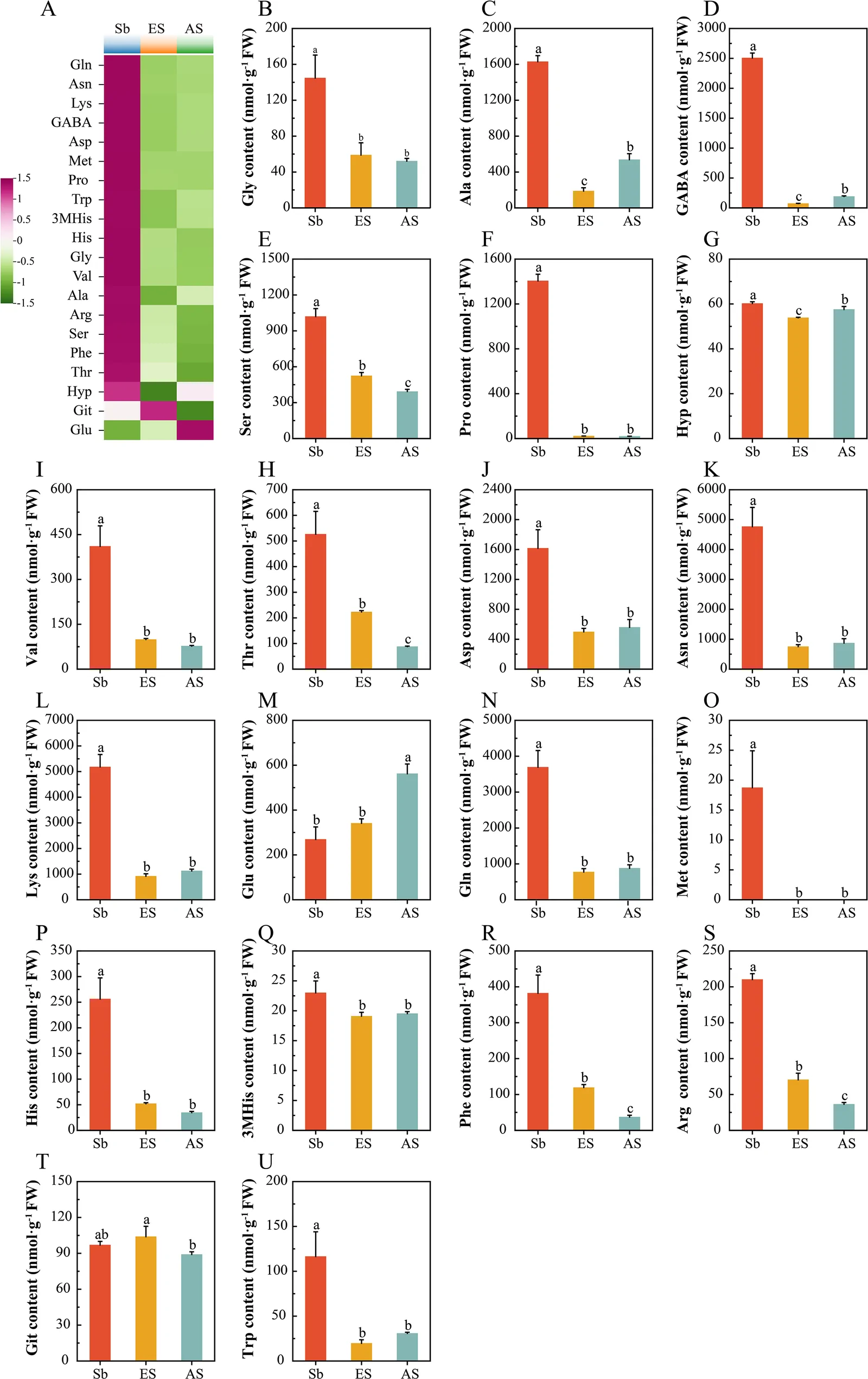
**Amino acid contents of *F. yunnanensis* shoots at different developmental stages.** (A) Heat map showing changes in amino acid contents at different growth stages. Data were *Z*-score standardized. The color gradient reflects the abundance of each amino acid across different developmental stages. Purple-red: higher relative content; green: lower relative content. (B) Gly content; (C) Ala content; (D) GABA content; (E) Ser content; (F) Pro content; (G) Hyp content; (H) Val content; (I) Thr content; (J) Asp content; (K) Asn content; (L) Lys content; (M) Glu content; (N) Gln content; (O) Met content; (P) His content; (Q) 3MHis content; (R) Phe content; (S) Arg content; (T) Cit content; (U) Trp content. Data are presented as mean ± SD from three biological replicates (*n* = 3). Statistical significance was assessed using one-way ANOVA followed by Duncan's multiple comparison test. Different lowercase letters indicate significant differences (*p* < 0.05) among growth stages. Sb, shoot buds; ES, normal edible bamboo shoots (30–50 cm in height); AS, aged bamboo shoots, whose first internode had completed elongation growth.

**Fig. S8:**
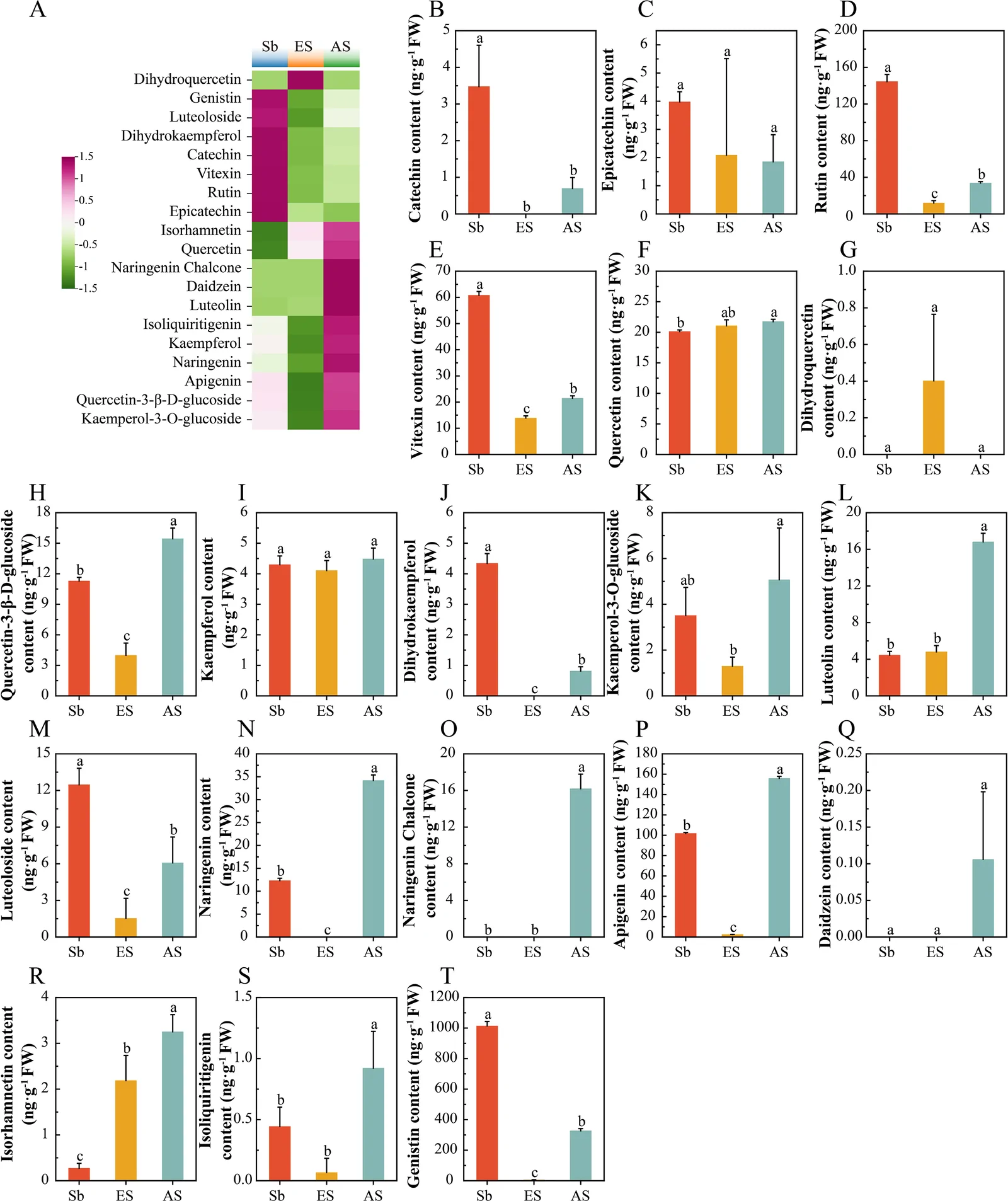
**Flavonoid contents of *F. yunnanensis* shoots at different developmental stages.** (A) Heat map showing changes in flavonoid contents at different growth stages. Data were *Z*-score standardized. The color gradient reflected the abundance of each flavonoid across different developmental stages. Purple-red: higher relative content; green: lower relative content. (B) Catechin content; (C) Epicatechin content; (D) Rutin content; (E) Vitexin content; (F) Quercetin content; (G) Dihydroquercetin content; (H) Quercetin-3-O-glucoside content; (I) Kaempferol content; (J) Dihydrokaempferol content; (K) Kaempferol-3-O-glucoside content; (L) Luteoloside content; (M) Luteolin content; (N) Naringenin content; (O) Naringin chalcone content; (P) Apigenin content; (Q) Isorhamnetin content; (R) Isoliquiritigenin content; (S) Daidzein content; (T) Genistin content. Data are presented as mean ± SD from three biological replicates (*n* = 3). Statistical significance was assessed using one-way ANOVA followed by Duncan's multiple comparison test. Different lowercase letters indicated significant differences (*p* < 0.05) among growth stages. Sb, shoot buds; ES, normal edible bamboo shoots (30–50 cm in height); AS, aged bamboo shoots, whose first internode had completed elongation growth.

**Fig. S9:**
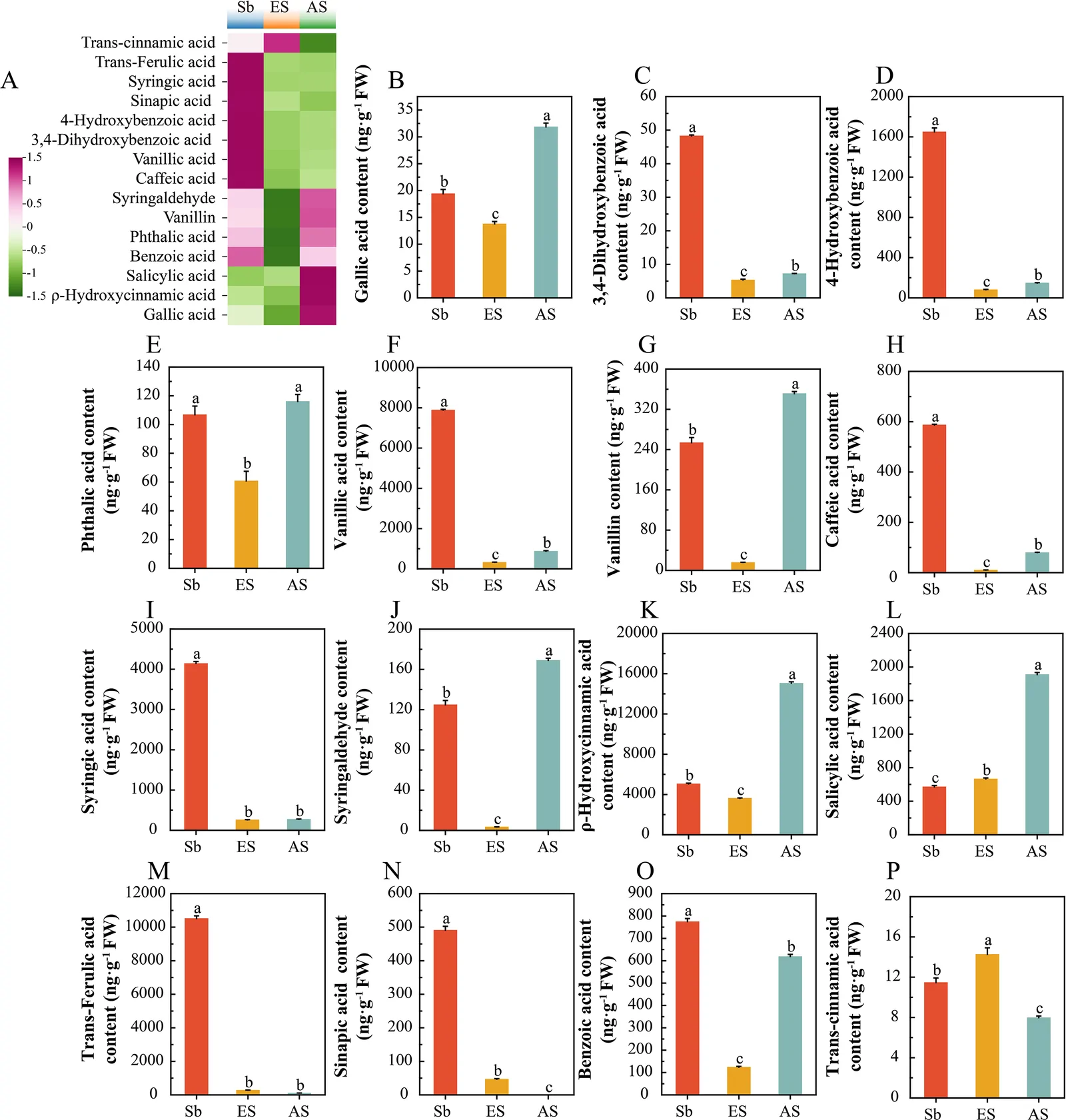
**Phenolic acid contents of *F. yunnanensis* shoots at different developmental stages.** (A) Heat map showing changes in phenolic acid contents at different growth stages. Data were *Z*-score standardized. The color gradient reflected the abundance of each phenolic acid across different developmental stages. Purple-red: higher relative content; green: lower relative content. (B) Gallic acid content; (C) 3,4-Dihydroxybenzoic acid content; (D) 4-Hydroxybenzoic acid content; (E) Phthalic acid content; (F) Vanillic acid content; (G) Vanillin content; (H) Caffeic acid content; (I) Syringic acid content; (J) Syringaldehyde content; (K) p-Hydroxycinnamic acid content; (L) Salicylic acid content; (M) Trans-Ferulic acid content; (N) Sinapic acid content; (O) Benzoic acid content; (P) Trans-Cinnamic acid content. Data are presented as mean ± SD from three biological replicates (*n* = 3). Statistical significance was assessed using one-way ANOVA followed by Duncan's multiple comparison test. Different lowercase letters indicated significant differences (*p* < 0.05) among growth stages. Sb, shoot buds; ES, normal edible bamboo shoots (30–50 cm in height); AS, aged bamboo shoots, whose first internode had completed elongation growth.

**Fig. S10:**
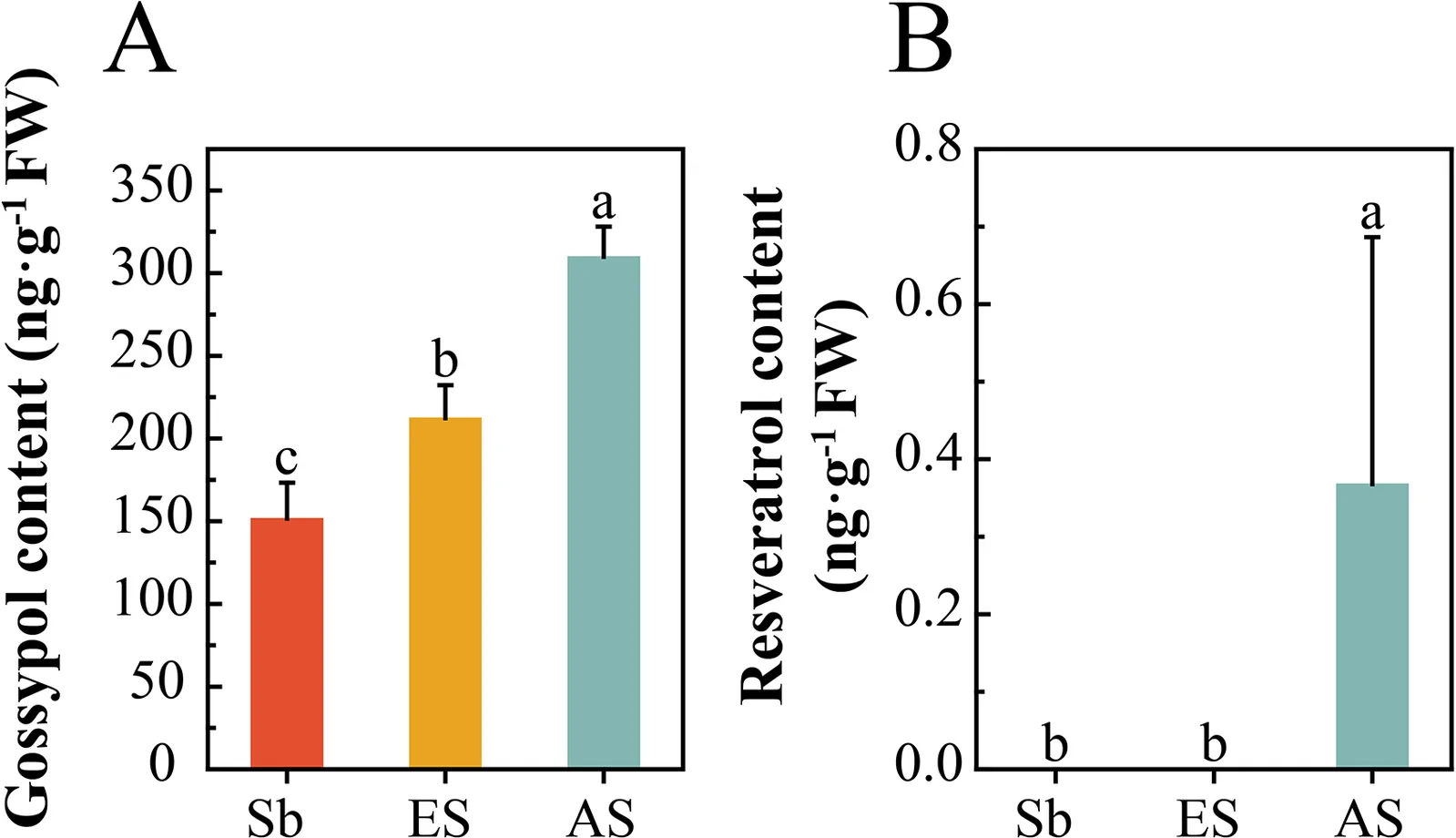
**Contents of other phenolic compounds in *F. yunnanensis* shoots at different growth stages.** (A) Gossypol content; (B) Resveratrol content. Data are presented as mean ± SD from three biological replicates (*n* = 3). Statistical significance was assessed using one-way ANOVA followed by Duncan's multiple comparison test. Different lowercase letters indicated significant differences (*p* < 0.05) among growth stages. Sb, shoot buds; ES, normal edible bamboo shoots (30–50 cm in height); AS, aged bamboo shoots, whose first internode had completed elongation growth.

**Fig. S11:**
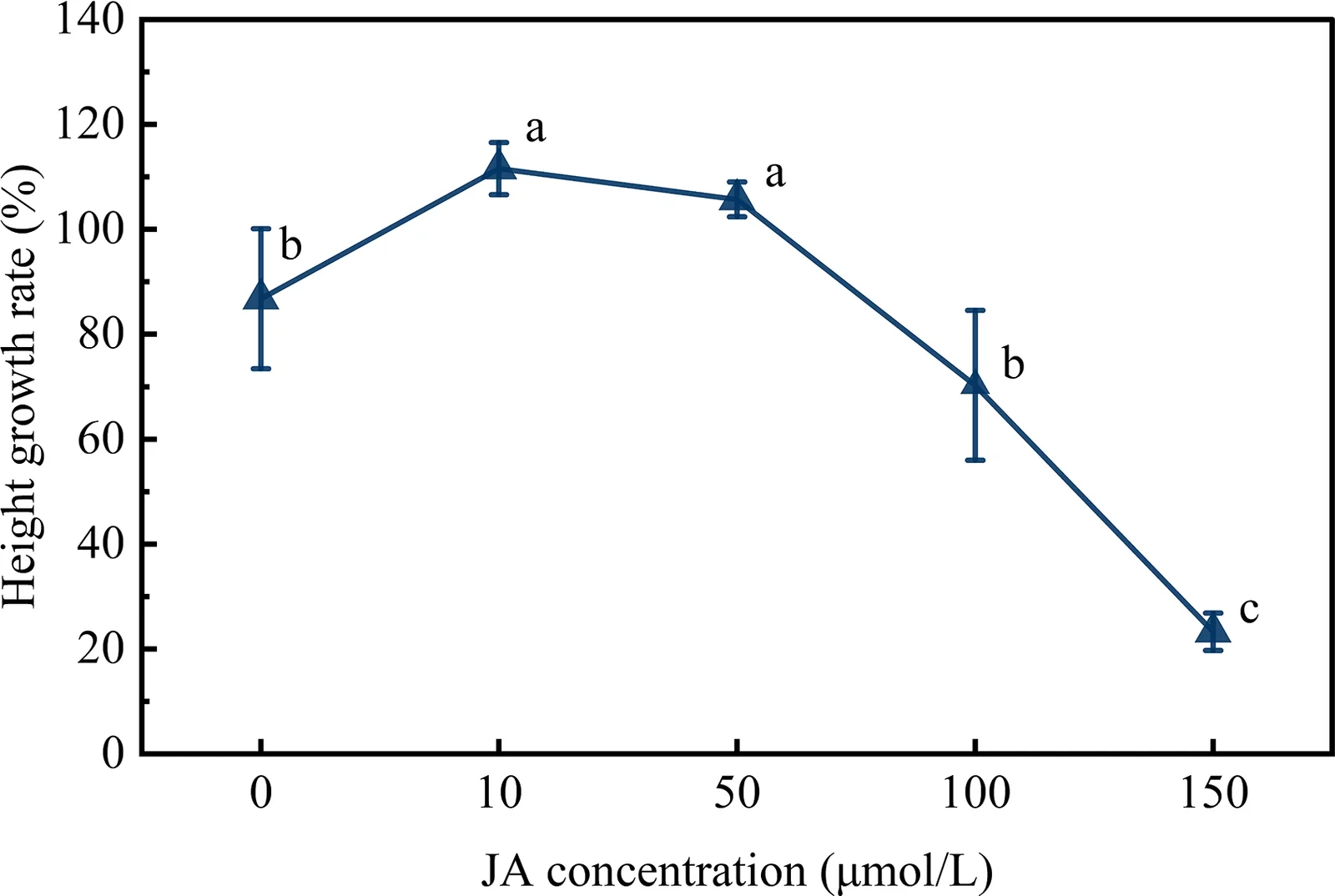
**Shoot height growth rate of *F. yunnanensis* shoots after 5 days of treatment with different JA concentrations.** Data are presented as mean ± SD from three biological replicates (*n* = 3). Statistical significance was assessed using one-way ANOVA followed by Duncan's multiple comparison test. Different lowercase letters indicated significant differences (*p* < 0.05) among different JA concentrations.

**Fig. S12:**
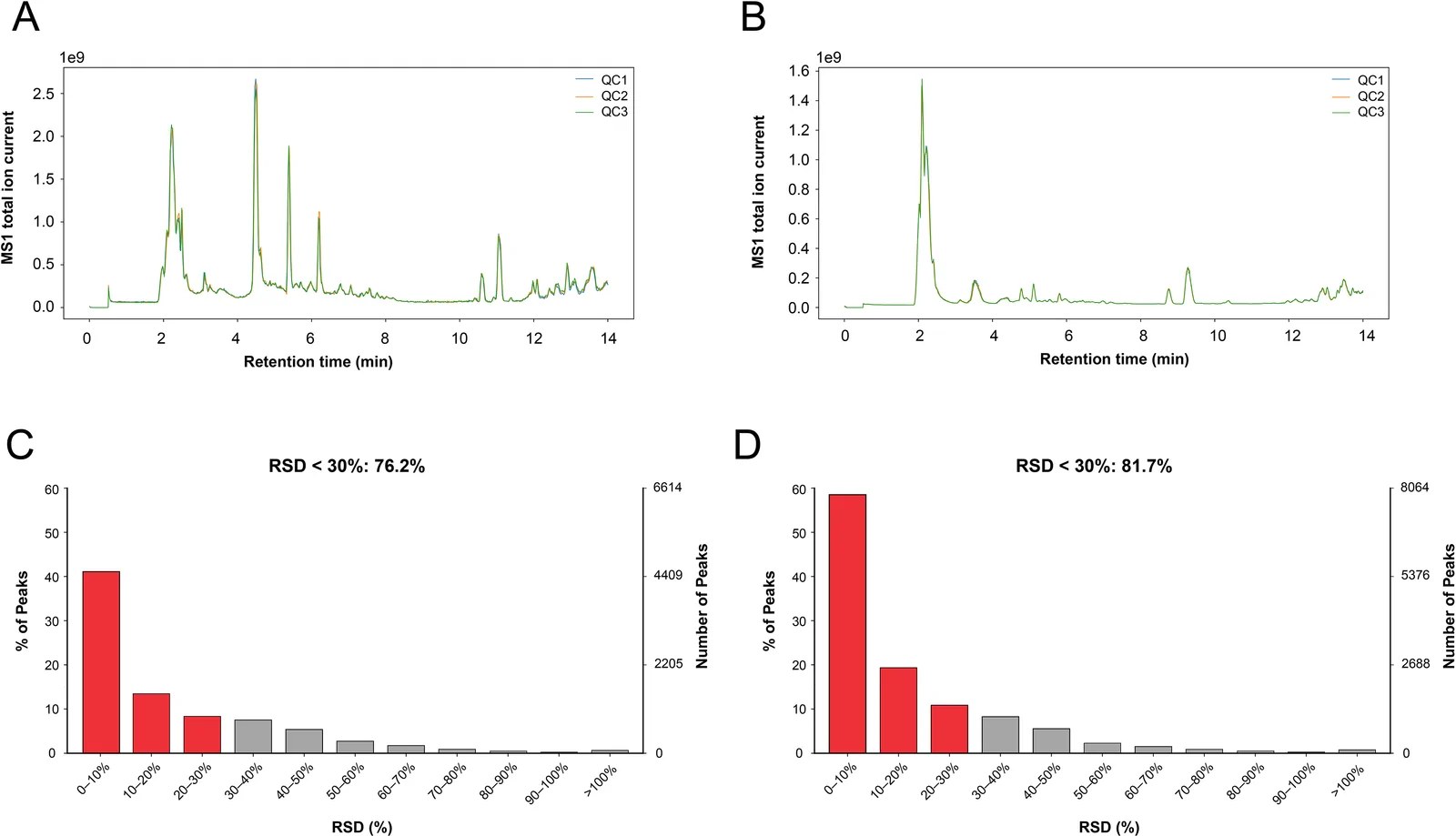
**Total ion chromatograms and relative standard deviation (RSD) distributions of QC samples for untargeted metabolomic analysis of bamboo shoots subjected to preharvest JA treatment.** (A, B) Total ion chromatograms in positive and negative ion modes, respectively. (C, D) RSD distributions of peak areas across QC samples in positive and negative ion modes, respectively. Red bars indicate RSD \ < 30%, accounting for 76.2% and 81.7% of the detected features, respectively.

**Fig. S13:**
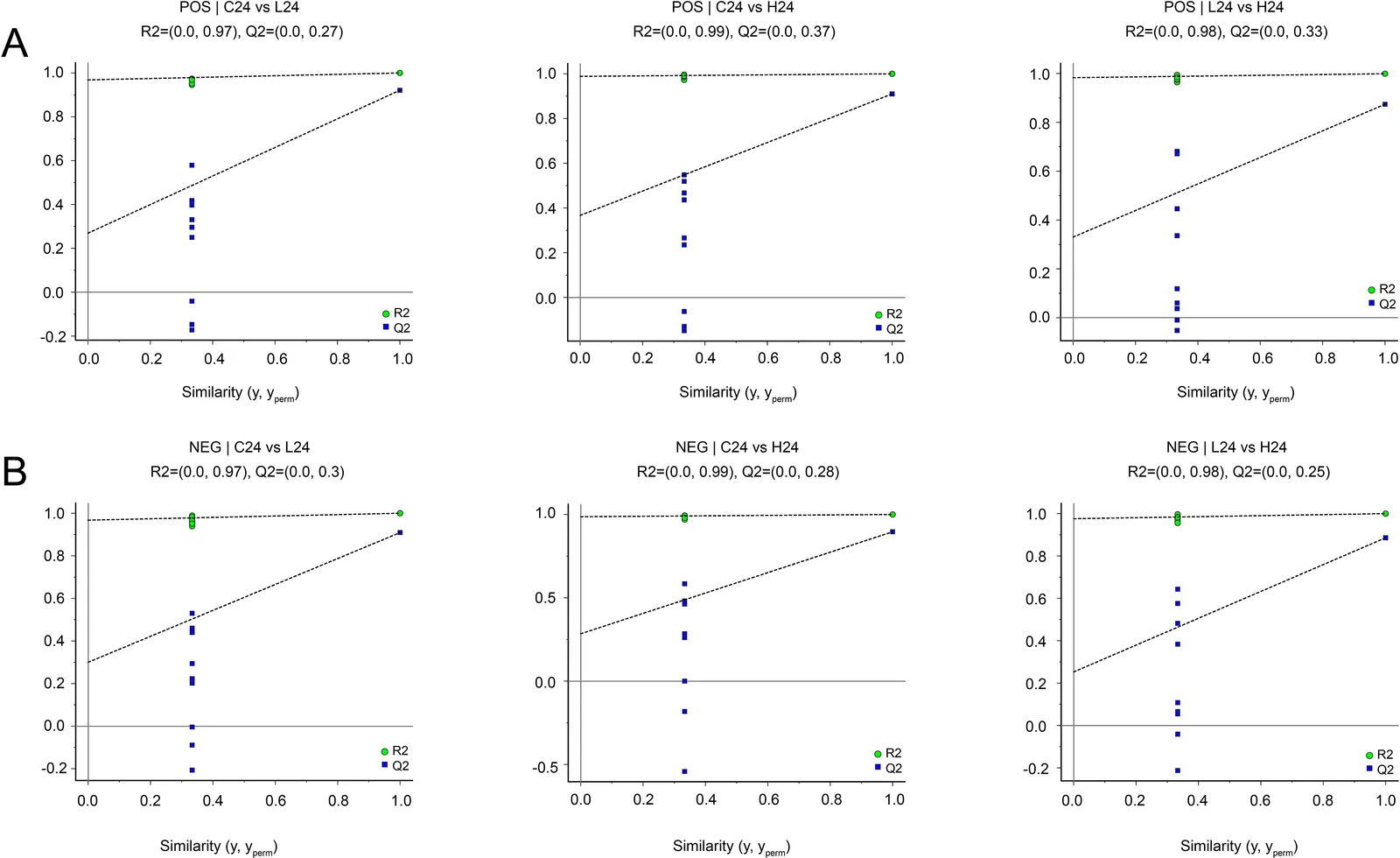
**Permutation tests of OPLS-DA models for bamboo shoots subjected to preharvest JA treatment.** (A) Positive ion mode. (B) Negative ion mode. Green circles and blue squares represent R^2^Y and Q^2^ values, respectively; dashed lines indicate linear regression fits.

**Fig. S14:**
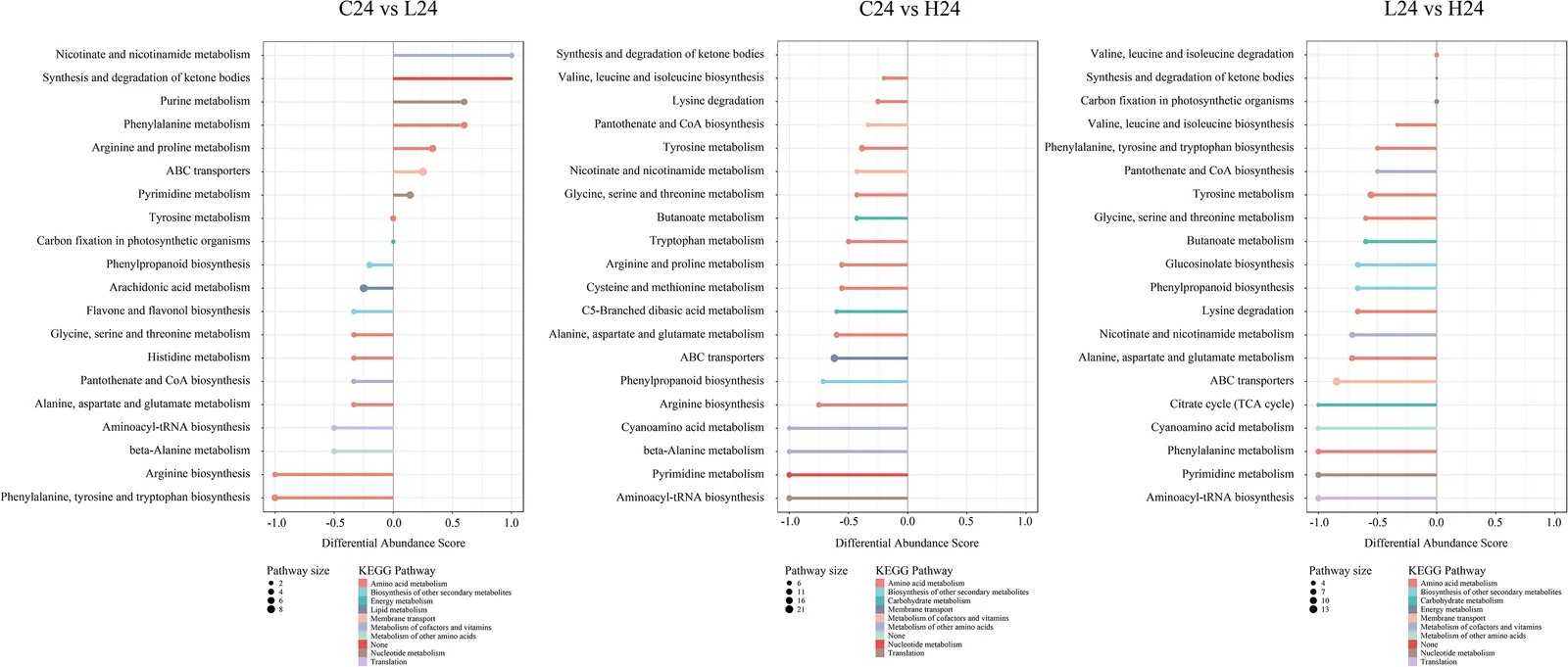
Pathway differential abundance scores for pairwise comparisons of JA treatments.

**Fig. S15:**
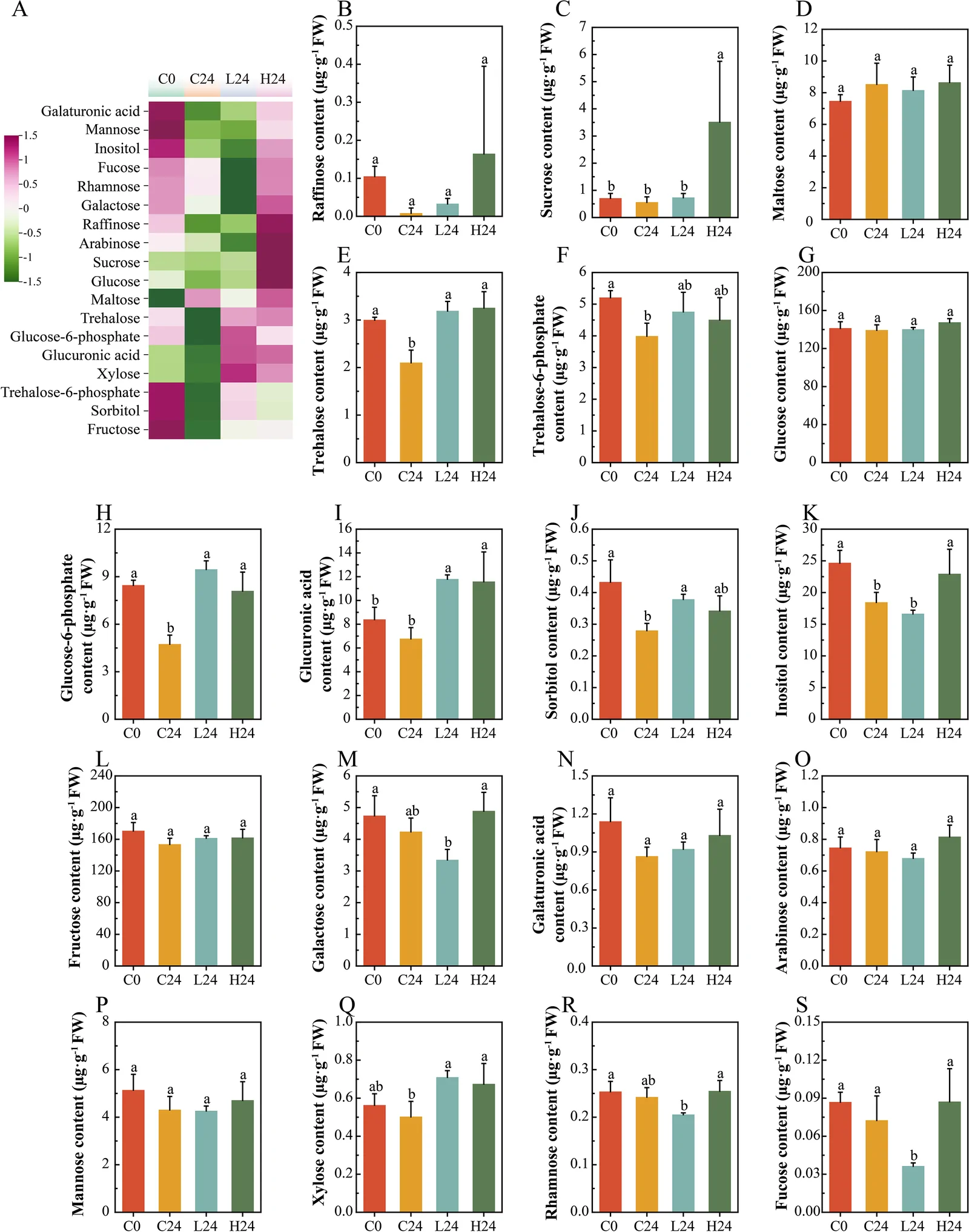
**Changes in soluble sugar contents of *F. yunnanensis* shoots after treatment with different JA concentrations.** (A) Heat map showing changes in soluble sugar contents under different JA concentrations. Data were *Z*-score standardized. The color gradient reflected the abundance of each soluble sugar across different treatments. Purple-red: increased relative content; green: decreased relative content. (B) Raffinose content; (C) Sucrose content; (D) Maltose content; (E) Trehalose content; (F) Trehalose-6-phosphate content; (G) Glucose content; (H) Glucose-6-phosphate content; (I) Glucuronic acid content; (J) Sorbitol content; (K) Inositol content; (L) Fructose content; (M) Galactose content; (N) Galacturonic acid content; (O) Arabinose content; (P) Mannose content; (Q) Xylose content; (R) Rhamnose content; (S) Fucose content. Data are presented as mean ± SD from three biological replicates (*n* = 3). Statistical significance was assessed using one-way ANOVA followed by Duncan's multiple comparison test. Different lowercase letters indicated significant differences (*p* < 0.05) among different JA concentrations. C0, untreated bamboo shoots (0 h); C24, bamboo shoots treated with lanolin (vehicle control) for 24 h; L24, bamboo shoots treated with low concentration of JA (10 μmol·L^−1^) for 24 h; H24, bamboo shoots treated with high concentration of JA (150 μmol·L^−1^) for 24 h.

**Fig. S16:**
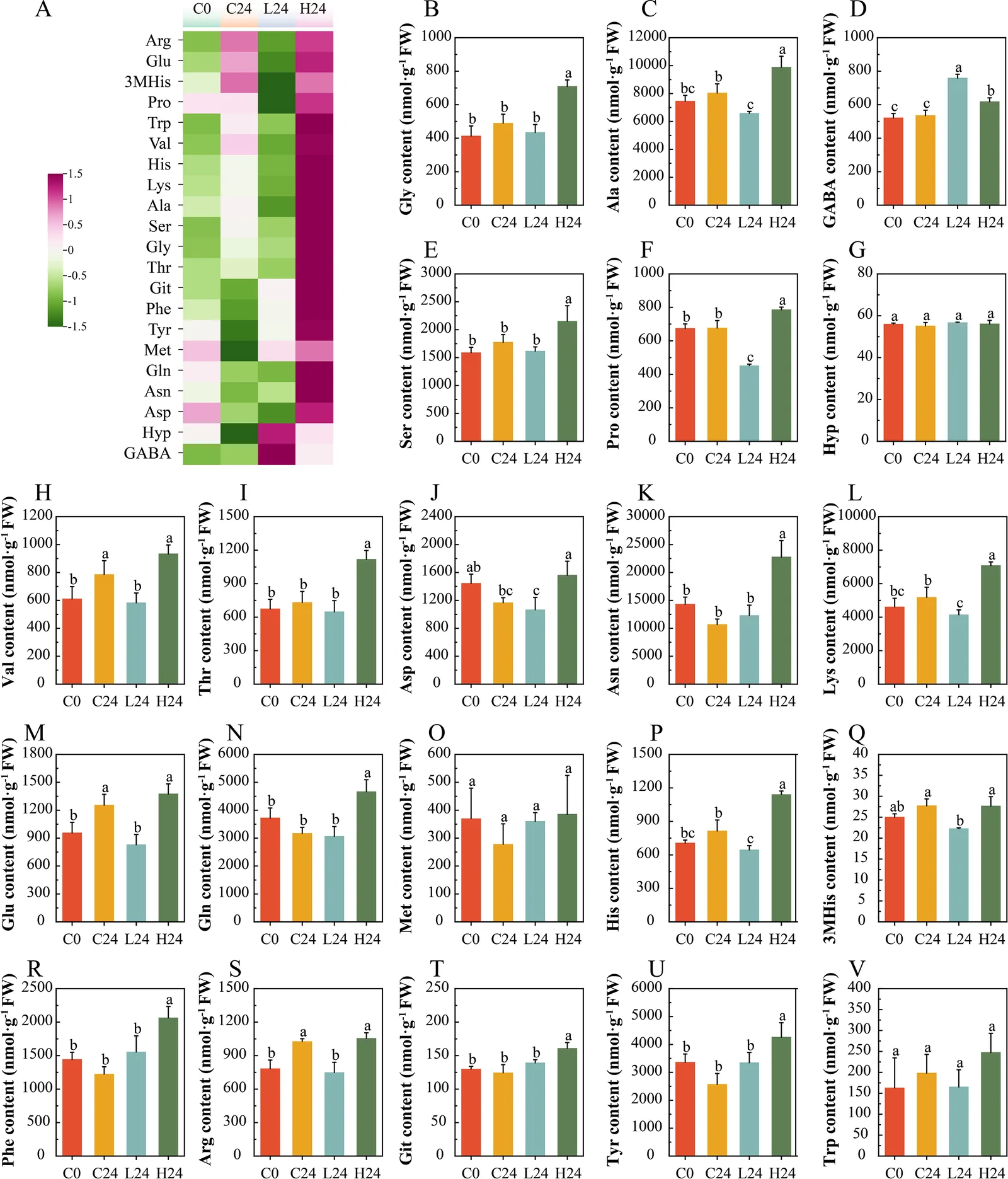
**Changes in amino acid contents of *F. yunnanensis* shoots after treatment with different JA concentrations.** (A) Heat map showing changes in amino acid contents under different JA concentrations. Data were Z-score standardized. The color gradient reflected the abundance of each amino acid across different treatments. Purple-red: increased relative content; green: decreased relative content. (B) Gly content; (C) Ala content; (D) GABA content; (E) Ser content; (F) Pro content; (G) Hyp content; (H) Val content; (I) Thr content; (J) Asp content; (K) Asn content; (L) Lys content; (M) Glu content; (N) Gln content; (O) Met content; (P) His content; (Q) 3MHis content; (R) Phe content; (S) Arg content; (T) Cit content; (U) Tyr content; (V) Trp content. Data are presented as mean ± SD from three biological replicates (*n* = 3). Statistical significance was assessed using one-way ANOVA followed by Duncan's multiple comparison test. Different lowercase letters indicated significant differences (*p* < 0.05) among different JA concentrations. C0, untreated bamboo shoots (0 h); C24, bamboo shoots treated with lanolin (vehicle control) for 24 h; L24, bamboo shoots treated with low concentration of JA (10 μmol·L^−1^) for 24 h; H24, bamboo shoots treated with high concentration of JA (150 μmol·L^−1^) for 24 h.

**Fig. S17:**
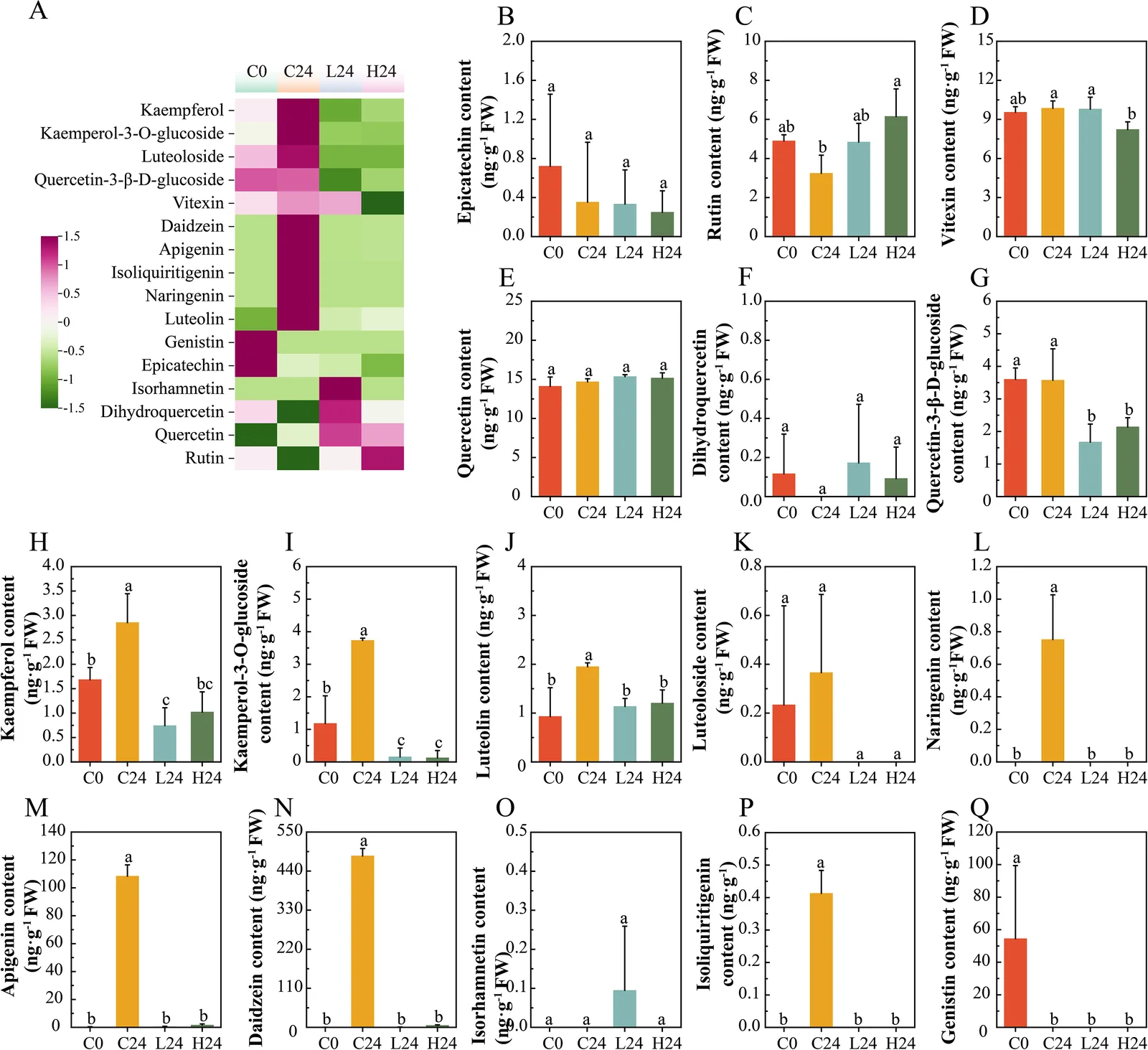
**Changes in flavonoid contents of *F. yunnanensis* shoots after treatment with different JA concentrations.** (A) Heat map showing changes in flavonoid contents under different JA concentrations. Data were Z-score standardized. The color gradient reflected the abundance of each flavonoid across different treatments. Purple-red: increased relative content; green: decreased relative content. (B) Epicatechin content; (C) Rutin content; (D) Vitexin content; (E) Quercetin content; (F) Dihydroquercetin content; (G) Quercetin-3-O-glucoside content; (H) Kaempferol content; (I) Kaempferol-3-O-glucoside content; (J) Luteolin content; (K) Luteoloside content; (L) Naringenin content; (M) Apigenin content; (N) Daidzein content; (O) Isorhamnetin content; (P) Isoliquiritigenin content; (Q) Genistin content. Data are presented as mean ± SD from three biological replicates (*n* = 3). Statistical significance was assessed using one-way ANOVA followed by Duncan's multiple comparison test. Different lowercase letters indicated significant differences (*p* < 0.05) among different JA concentrations. C0, untreated bamboo shoots (0 h); C24, bamboo shoots treated with lanolin (vehicle control) for 24 h; L24, bamboo shoots treated with low concentration of JA (10 μmol·L^−1^) for 24 h; H24, bamboo shoots treated with high concentration of JA (150 μmol·L^−1^) for 24 h.

**Fig. S18:**
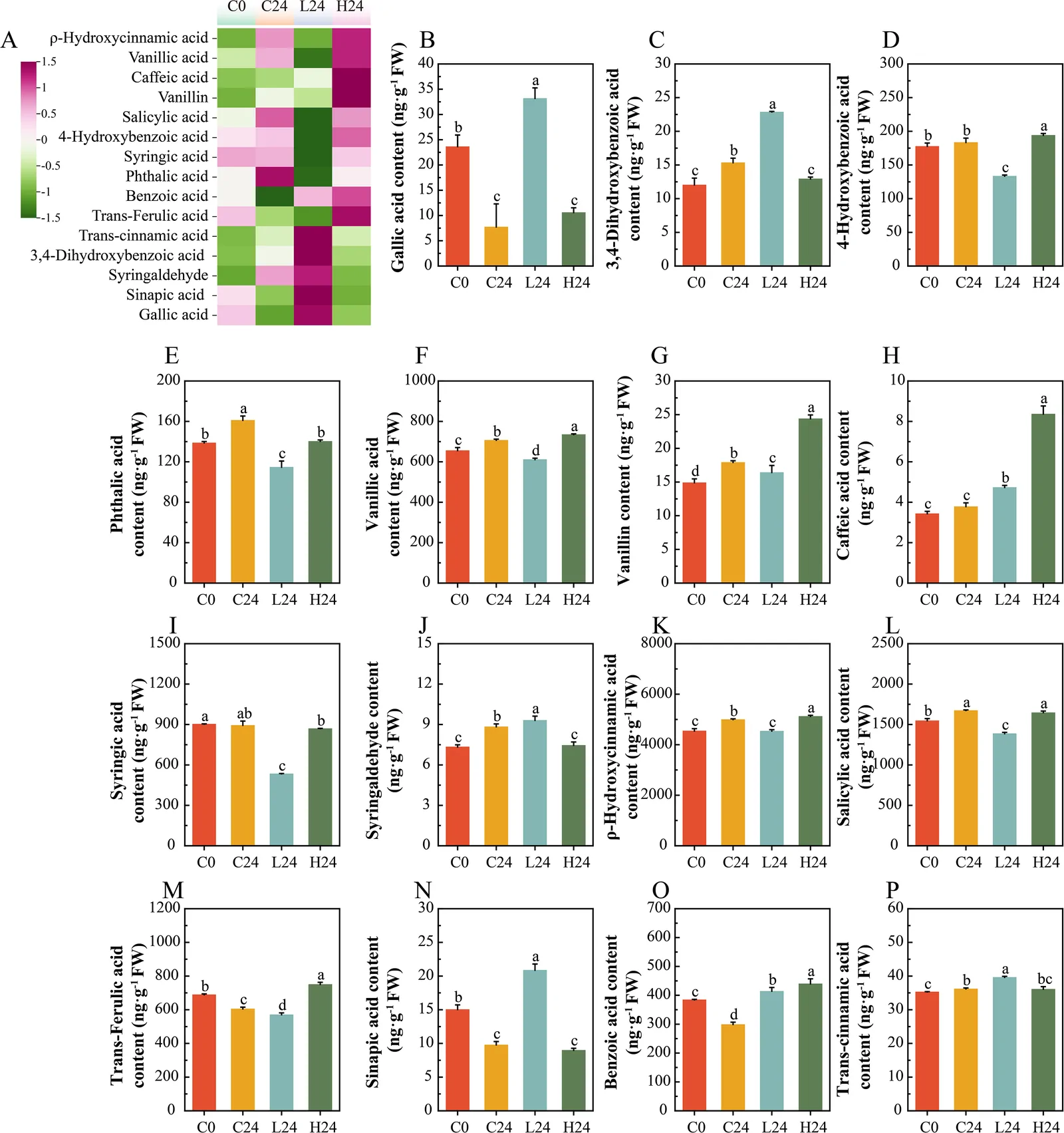
**Changes in phenolic acid contents of *F. yunnanensis* shoots after treatment with different JA concentrations.** (A) Heat map showing changes in phenolic acid contents under different JA concentrations. Data were Z-score standardized. The color gradient reflected the abundance of each phenolic acid across different treatments. Purple-red: increased relative content; green: decreased relative content. (B) Gallic acid content; (C) 3,4-Dihydroxybenzoic acid content; (D) 4-Hydroxybenzoic acid content; (E) Phthalic acid content; (F) Vanillic acid content; (G) Vanillin content; (H) Caffeic acid content; (I) Syringic acid content; (J) Syringaldehyde content; (K) p-Hydroxycinnamic acid content; (L) Salicylic acid content; (M) trans-Ferulic acid content; (N) Sinapic acid content; (O) Benzoic acid content; (P) trans-Cinnamic acid content. Data are presented as mean ± SD from three biological replicates (*n* = 3). Statistical significance was assessed using one-way ANOVA followed by Duncan's multiple comparison test. Different lowercase letters indicated significant differences (*p* < 0.05) among different JA concentrations. C0, untreated bamboo shoots (0 h); C24, bamboo shoots treated with lanolin (vehicle control) for 24 h; L24, bamboo shoots treated with low concentration of JA (10 μmol·L^−1^) for 24 h; H24, bamboo shoots treated with high concentration of JA (150 μmol·L^−1^) for 24 h.

**Fig. S19:**
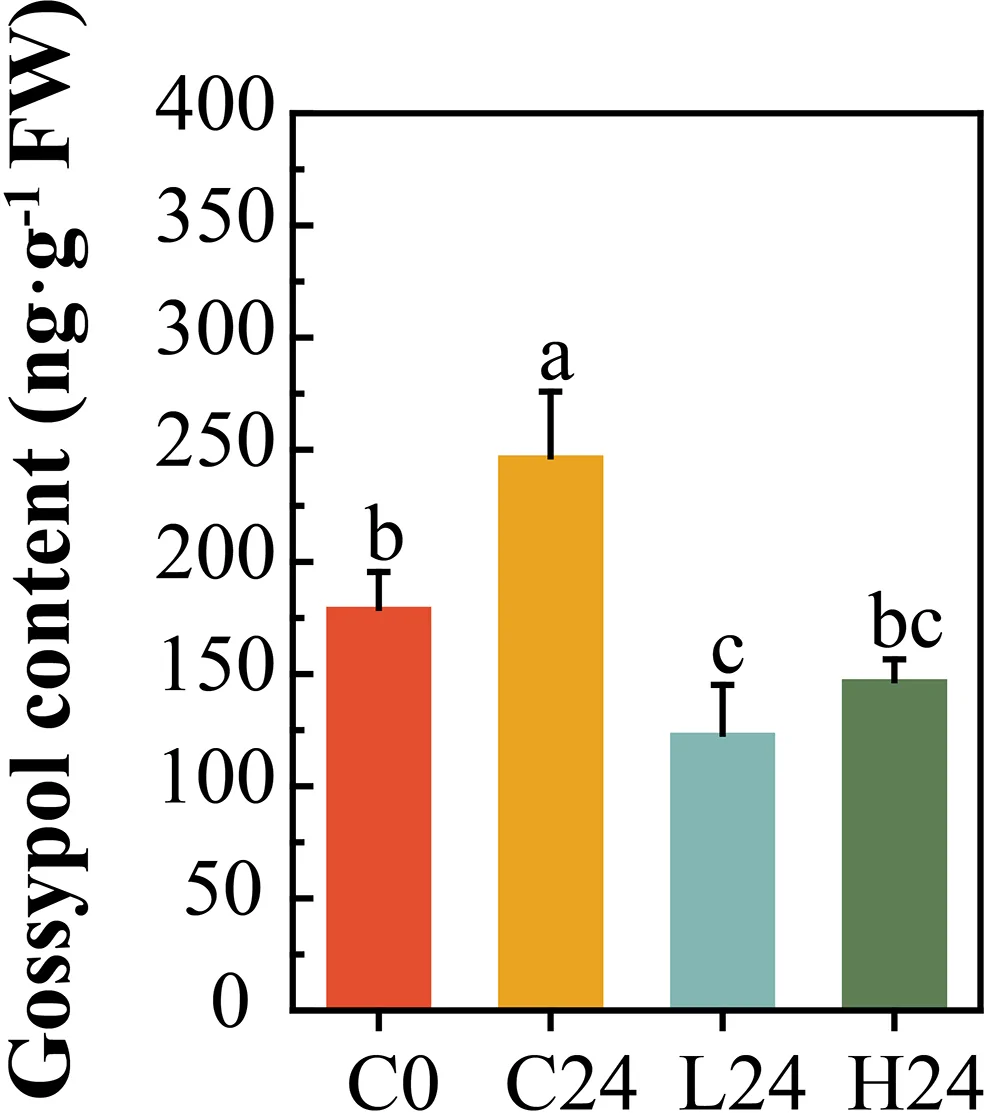
**Changes in gossypol content in *F. yunnanensis* shoots after treatment with different JA concentrations.** Data are presented as mean ± SD from three biological replicates (*n* = 3). Statistical significance was assessed using one-way ANOVA followed by Duncan's multiple comparison test. Different lowercase letters indicated significant differences (*p* < 0.05) among different JA concentrations. C0, untreated bamboo shoots (0 h); C24, bamboo shoots treated with lanolin (vehicle control) for 24 h; L24, bamboo shoots treated with low concentration of JA (10 μmol·L^−1^) for 24 h; H24, bamboo shoots treated with high concentration of JA (150 μmol·L^−1^) for 24 h.

Supplementary material Tables S1-S11.

## Author contribution

Jiaxin Liu performed part of the experiments, analyzed the data, and drafted the manuscript. Yingdan Yan collected the samples and completed most of the experiments. Wanli Shi, Bo Zheng, Fangwei Zhu, Ye Chen, and Jiaxin Ran collected and processed the samples. Lingfeng Li, Lixia Yu, Huijin Fan, and Jialong Pei supervised the experiments. Shuguang Wang designed the project, provided supervision, and participated in writing the manuscript. All authors read and approved the final manuscript.

## CRediT authorship contribution statement

**Jiaxin Liu:** Writing – original draft, Visualization, Software, Resources, Methodology, Investigation, Formal analysis, Data curation, Conceptualization. **Yingdan Yan:** Resources, Methodology, Investigation. **Wanli Shi:** Resources, Methodology, Investigation. **Fangwei Zhu:** Resources, Investigation. **Bo Zheng:** Resources, Investigation. **Lingfeng Li:** Supervision. **Ye Chen:** Investigation. **Jiaxin Ran:** Investigation. **Lixia Yu:** Supervision. **Huijin Fan:** Supervision. **Jialong Pei:** Supervision. **Shuguang Wang:** Writing – review & editing, Supervision, Resources, Project administration, Methodology, Data curation, Conceptualization.

## Funding

This paper was funded by the Yunnan Provincial Key Area Science and Technology Plan Project (No. 202602AE090069), 10.13039/501100005273Natural Science Foundation of Yunnan Province (202201AS070018), and National Natural Science Fund of China (32460376).

## Declaration of competing interest

The authors declare that they have no known competing financial interests or personal relationships that could have appeared to influence the work reported in this paper.

## Data Availability

Data will be made available on request.
